# Divergent host–pathogen interactions in neurolisteriosis: cytosolic replication vs. phagosomal dormancy of *Listeria monocytogenes* in CNS macrophages

**DOI:** 10.1007/s00401-025-02900-8

**Published:** 2025-06-16

**Authors:** Leticia Tavares-Gomes, Margherita Polidori, Camille Monney, Géraldine Neuhaus, Beatriz Vidondo, Guillaume Witz, Andrew Hemphill, Anna Oevermann

**Affiliations:** 1 Division of Neurological Sciences, Department of Clinical Research and Veterinary Public Health, Vetsuisse Faculty, University of Bern, Bern, Switzerland; 2 Graduate School for Cellular and Biomedical Sciences, University of Bern, Bern, Switzerland; 3 Present Address: i3S – Institute for Research and Innovation in Health, University of Porto, Porto, Portugal; 4 Veterinary Public Health Institute, University of Bern, Bern, Switzerland; 5 Data Science Lab, University of Bern, Bern, Switzerland; 6 Institute of Parasitology, Vetsuisse Faculty, University of Bern, Bern, Switzerland

**Keywords:** Neurolisteriosis, Microglia, Monocyte-derived macrophages, Viable but non-culturable (VBNC) bacteria, Persistence

## Abstract

Bacterial infections of the central nervous system (CNS) pose a significant threat to public health, especially with the growing challenge of antimicrobial resistance. Among these, *Listeria monocytogenes* (*Lm*) stands out as a key pathogen, responsible for often fatal neurolisteriosis in humans and cattle. Emerging evidence highlights the distinct roles played by microglia, the resident macrophages of the CNS, and infiltrating monocyte-derived macrophages (MDM) during neuroinflammation. Using bovine models, we investigated the interactions between these two macrophage populations and *Lm* during infection. Our results show that *Lm* thrives in the cytosol of microglia, driving productive infection and facilitating bacterial spread. In contrast, MDM effectively sequesters *Lm* within the phagolysosomal system, limiting its replication and inducing a viable but non-culturable (VBNC) state without completely eliminating the pathogen. Listeriolysin O contributes to the dichotomy of *Lm* fate, determining whether *Lm* escapes into the cytosol or transitions to the VBNC state. These findings underscore the complexity of *Lm*-host dynamics in neurolisteriosis, emphasizing the distinct yet complementary roles of microglia and MDM in shaping CNS infection. By elucidating these mechanisms, our study offers new perspectives on the neurolisteriosis pathogenesis and opens avenues for innovative therapeutic approaches to combat bacterial neuroinfections.

**Supplementary Information:**

The online version contains supplementary material available at 10.1007/s00401-025-02900-8.

## Introduction

*Listeria monocytogenes* (*Lm*) is a Gram-positive bacterium renowned for its remarkable adaptability to different habitats. As a result, it is widespread in the environment and able to cause disease in a large variety of mammalian and non-mammalian species, including humans and ruminants [14, 65]. Upon ingestion by the host, *Lm* can colonize the gastrointestinal tract, where the bacterium may cause mild gastroenteritis. However, under certain circumstances and in vulnerable populations such as in neonates, the elderly, pregnant women, and immunosuppressed individuals, *Lm* can cross the gastrointestinal barrier and cause severe clinical forms, including septicemia, miscarriage, and frequently fatal neuroinfections known as neurolisteriosis [65].

Neurolisteriosis poses a serious concern due to its high mortality rate in both humans and ruminants despite antibiotic treatment. In humans, it ranks among the leading causes of meningoencephalitis [66, 70] and in ruminants it is one of the most common neuroinfections, frequently causing outbreaks on farms and substantial economic losses [6, 56]. In addition, ruminants may represent an on-farm reservoir of *Lm* for human infection via contamination of dairy products [6, 49, 56]. However, our understanding of the mechanisms underlying host–pathogen interaction during neurolisteriosis remains limited.

Both humans and ruminants afflicted with the rhombencephalitic form of neurolisteriosis exhibit marked inflammation in the brainstem, characterized by mononuclear perivascular cuffs and pathognomonic microabscesses, which are aggregates of different phagocytic cell types including resident brain macrophages (microglia) and macrophages infiltrating from the periphery [18, 55, 76]. Notably, during the acute phase of neurolisteriosis, these phagocytes contain high numbers of bacteria [55], raising questions about the role of phagocyte subpopulations in supporting bacterial growth versus eliminating the pathogen.

Although macrophages, well-equipped to detect, engulf and kill pathogens, are traditionally viewed as key effector cells in the fight against infection, several studies indicate that macrophage populations diverge in their ability to kill *Lm*, which possibly depends on the macrophage activation status and tissue origin [12, 20, 54, 64, 73, 78, 80]. Different phagocytic pathways are involved in the uptake of *Lm* by macrophages. During conventional phagocytosis, *Lm* can undergo two fates: either it remains trapped in a vacuole and is subsequently killed, or it escapes the vacuole into the cytosol. While the former is a critical step of bacterial killing via phagolysosomal fusion in macrophages [13, 58, 73, 82], the latter occurs when *Lm* rapidly exits the phagocytic vacuole following phagosomal acidification through the combined action of a potent pore-forming toxin, listeriolysin-O (LLO), and two phospholipases C (PlcA and PlcB) before phagolysosomal fusion occurs. Vacuolar escape allows *Lm* to complete its canonical life cycle by replicating in the cytosol and spreading to other cells through actA-mediated host actin polymerization [62, 81]. However, if *Lm* is insufficiently protected by polymerized actin, it can be engulfed by the host cell’s canonical autophagic machinery, leading to its death [37].

While the fate of *Lm* during conventional phagocytosis is well established, novel intracellular lifestyles of *Lm* have been associated with unconventional phagocytic and autophagic pathways. In epithelial cells, *Lm* can exploit the autophagy system to survive in large vacuoles known as *Listeria*-Containing vacuoles (LisCVs) [41]. Within these LisCVs, *Lm* persists in different physiological states, including slowly replicating bacteria and viable but non-culturable (VBNC) forms [41], the latter being a bacterial survival strategy that allows the pathogen to withstand adverse conditions until the environment becomes more favourable [48]. In addition, *Lm* may utilize an unconventional phagocytic pathway, LC3-associated phagocytosis (LAP), to establish an intracellular proliferative niche within spacious *Listeria*-containing phagosomes (SLAP) [11, 59, 77]. However, this pathway has also been implicated in the killing of *Lm* by peritoneal macrophages [28, 36], highlighting the complex dynamics of *Lm*'s interaction with host cells. Interestingly, some macrophage populations support *Lm* vacuolar escape and proliferation, while others effectively kill the pathogen following vacuolar entrapment [20, 24, 25, 31, 63]. In addition, individual *Lm* within the same macrophage may exhibit different fates, suggesting that bacterial factors significantly contribute to the outcome of macrophage infection [52]. While there is a large body of literature on the fate of *Lm* in macrophages, little is known about its interaction with microglia, the resident macrophages in the CNS [16, 25, 32]. During steady state, microglia are the only macrophage population in the healthy brain parenchyma [43, 75]. In contrast, during neuroinflammation [56], the neuroparenchymal immune landscape can change dramatically as monocytes—alongside with other leukocytes—cross the blood brain barrier (BBB) and differentiate into monocyte-derived macrophages (MDM) [19]. There is growing evidence that resident macrophages and infiltrating MDM may adopt different functions [15, 47, 72, 83]. Despite their involvement in neurolisteriosis-associated microabscesses, the specific interaction of microglia and infiltrating MDM with *Lm* remains poorly understood [35, 55]. This knowledge gap is partly due to the historical inability to distinguish microglia from infiltrating MDM during neuroinflammation. To address this gap, our group employed previously established markers to distinguish microglia from invading MDM in situ [76], using brain tissue from clinical cases of bovine neurolisteriosis archived in our brain bank. Furthermore, we used in vitro models of primary bovine microglia and MDM [76] as surrogates to dissect *Lm* infection dynamics in these two major phagocyte populations. Here, we show that the overall fate of *Lm* depends on the macrophage population it infects. While both microglia and MDM are able to kill a small subpopulation of intravacuolar *Lm*, microglia allow larger numbers of bacteria to escape into the cytosol, leading to exponential intracellular bacterial growth. In contrast, MDM keep bacteria in check by confining the vast majority of *Lm* to vacuoles. However, MDM fail to completely eliminate infection because *Lm* rapidly enters a VBNC state. Deletion of LLO induces VBNC *Lm* in microglia and enhances early bacterial clearance in MDM, indicating that LLO is a key virulence factor for bacterial fate in the macrophage, determining whether bacteria escape into the cytosol, persist as VBNC forms or die within vacuoles.

## Material and methods

### In situ immunofluorescence of neurolisteriosis cases

Immunofluorescence (IF) was performed on formalin-fixed paraffin-embedded (FFPE) brain sections from naturally infected cattle (n = 5) exhibiting inflammatory lesions with pathognomonic microabscesses characteristic of listeriosis [55]. Two consecutive slides were stained with different antibody combinations to identify microglia and MDM. Microglia were defined as P2RY12^+^ or IBA-1^+^ F13A1^−^ cells, while MDM were defined as P2RY12^−^ or IBA-1^+^ F13A1^+^ cells. P2RY12 and F13A1 were previously validated as specific markers for bovine microglia and MDM, respectively [76]. The first slide was stained with antibodies against P2RY12 [76] (rabbit anti-mouse; 1:200; AS-55043A, Anaspec), *Listeria* O antiserum [35] (rabbit; 1:200; BD, Allschwil, Switzerland) and diamidino-2-phenylindole (DAPI: 1:1000; Thermo Fisher Scientific). The consecutive slide was stained with an anti-F13A1 mouse antibody [76] (1:100; MA5-11751, Thermo Fisher Scientific), anti-IBA-1 rabbit antibody (a pan-macrophage marker; 1:500; 013-27593, Wako), *Listeria O* antiserum and DAPI. Secondary anti-mouse and anti-rabbit antibodies (Alexa Fluor 555 or Alexa-Fluor 647, Invitrogen) were applied at a dilution of 1:500 and incubated for 1 hour (h) at room temperature. To allow co-staining with additional rabbit primary antibodies, the *Listeria O* antiserum was pre-labelled using the Zenon™ Alexa Fluor 488 Rabbit IgG Labelling Kit (Z25302, Invitrogen), according to the manufacturer’s instructions. The labelled antibody was applied to the tissue slides for 30 minutes (min) at room temperature following secondary antibody incubation for detection of the other primary antibodies.

**Fig. 1:**
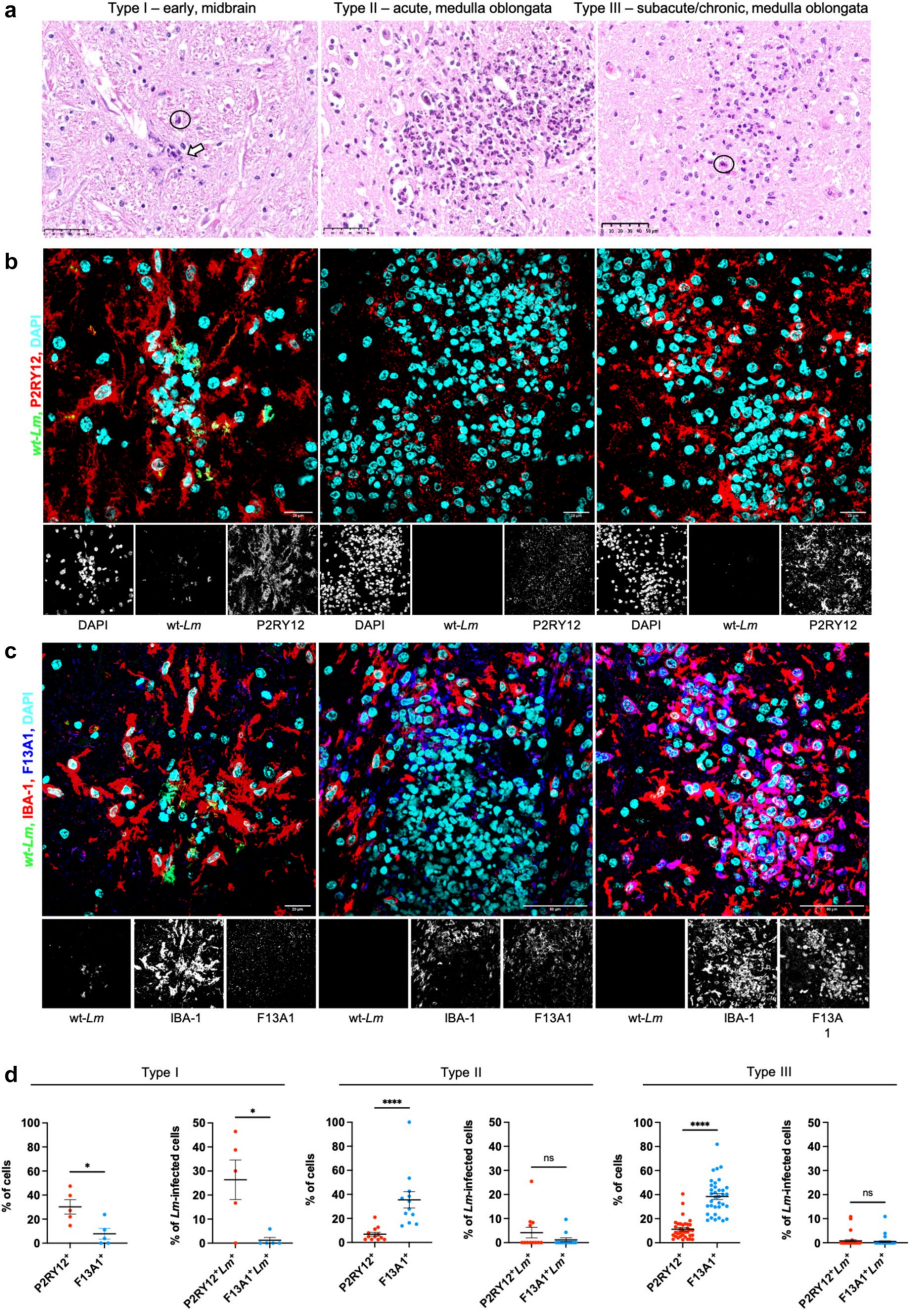
Microglia: a niche for *Listeria monocytogenes* replication in early microabscesses. Immunofluorescence analysis of microabscesses in bovine brains affected by neurolisteriosis using antibodies against *Lm*, the microglial protein P2RY12, the MDM protein F13A1 and the universal macrophage marker IBA-1. **a** H&E images of early (type I), acute (type II) and subacute/chronic (type III) microabscesses in the brainstem of cattle with neurolisteriosis, corresponding to the ones shown in the immunofluorescence images. Type I microabscesses are small, typically composed of a few activated microglia-like cells in close proximity to bacterial colonies (white arrow), along with scattered polymorphonuclear neutrophils (circled). Type II microabscesses are larger and predominantly composed of neutrophils, while type III microabscesses feature a dominant population of mononuclear MDM-like cells and scattered neutrophils (circled). **b** In early microabscesses (left panel), numerous *Lm* (green) are closely associated with P2RY12-expressing microglia (red), which constitute the primary cell type present. With progression of microabscesses (middle and right panels), infiltration by a greater number of P2RY12-negative cells occurs, coinciding with a decrease of bacterial burden. **c** Within early microabscesses (left panel), numerous *Lm* are associated with IBA1-positive and F13A1-negative cells (interpreted as microglia), while F13A1-positive MDM are sparsely detected. As microabscesses mature (middle and right panels), an increase in F13A1 positive cells coincides with a decrease in *Lm* number. **d** Percentage of P2RY12-positive microglia and F13A1-positive MDM among nucleated cells (left panel), as well as percentage of *Lm*-infected cells among P2RY12-positive microglia and F13A1-positive MDM (right panel) in early (type I, n = 5), acute (type II, n = 12), and subacute/chronic (type III, n = 35) microabscesses. The highest proportion of infected cells is observed among P2RY12-positive microglia, particularly evident in early microabscesses. Error bars represent SEM, with significance levels indicated as ns (not significant), * p < 0.05, and **** p < 0.0001 (Mann–Whitney U test)

In total, 52 microabscesses were analyzed. Histologic criteria for classification into type I (early microabscesses), type II (acute microabscesses) and type III (subacute to chronic microabscesses) in hematoxylin and eosin stained sections were as follows: (i) small size microabscesses consisting of very few phagocytes including rod-shaped microglia-like cells and single polymorphonuclear nuclei (neutrophils) for type I microabscesses, (ii) larger size with abundance of neutrophils in type II microabscesses, and (iii) larger size with predominance of densely packed round, MDM-like cells mixed with few neutrophils in type III microabscesses (Fig. 1). Image acquisition and analysis are described below.

### Isolation of primary bovine microglia and monocytes

Brain tissue and blood from calves slaughtered for human consumption were obtained from local abattoirs. When possible, both microglia and monocytes were isolated from the same animal. Brain tissue was processed immediately after harvesting or stored in Hibernate-A medium (Gibco, Life Technologies) at 4 °C for a maximum of 24 h until further processing. Isolation of microglia and peripheral blood mononuclear cells (PBMC) was performed as described in a previous study [76]. Briefly, microglia were obtained by mechanical dissociation followed by a single 37% Percoll gradient. For PBMC isolation, the buffy coat resulting from the first centrifugation was overlaid on an equal amount of Histopaque-1077 (Sigma-Aldrich) and the interface containing the PBMC was collected. The primary cells used in this study were previously characterized by RNA-sequencing [76]. Microglia and MDM showed high purity and expression of lineage specific markers.

### Culture of microglia and CD14^+^ MDM

Microglia were seeded in culture plates immediately after isolation. Monocytes were cultured after CD14^+^ selection by magnetic sorting (Milteny Biotec). Both myeloid cell types were cultured under the same conditions in 24 well plates (2.5 × 10^5^ cells per well) or 96 well plates (2.5 × 10^4^ cells per well). Cells were cultured in DMEM (Invitrogen, Gibco) supplemented with 10% FCS, 1% penicillin–streptomycin (P/S), and recombinant bovine macrophage colony-stimulating factor (M-CSF) (25 ng/mL, Kingfisher, Biotech, inc.). The medium was changed after 2 and 4 days in culture to remove detached cells and debris. Following 1 week in culture, the cells were used for bacterial infection.

### Bacterial strains

*Listeria monocytogenes (Lm)* strain JF5203 (NCBI Reference Sequence: NZ_ LT985474.1; https://www.ncbi.nlm.nih.gov/nuccore/NZ_LT985474.1), isolated from a case of bovine rhombencephalitis, was used as wildtype (wt). GFP-expressing Δ*hly* and Δ*actA* mutants of JF5203 were generated in previous studies [4, 35]. Δ*inlA*-*Lm* and Δ*inlB*-*Lm* were generated as an in-frame deletion mutants from the parental *Lm* strain JF5203. For the generation of *inlA* and *inlB* deletion mutants, either pMAD (*inlA*) [3] or pHOSS1 (*inlB*) [1] were used. DNA extracted from JF5203-wt was used as the template for the amplification of the up- and downstream regions of *inlA* and *inlB* by PCR (Roche, Basel, Switzerland) using the primer pairs 1 and 2 or 3 and 4 (SI Appendix, Table S2), respectively, with the Expand High Fidelity Plus PCR system (Roche Diagnostics, Rotkreuz, Switzerland). Resulting PCR products were cloned into pMAD or pHOSS1 using restriction enzymes SalI and XmaI. The two amplicons were connected by splice over extension PCR using primers 1 and 4 (SI Appendix, Table S2). Deletion of *inlA* and *inlB* was performed as described elsewhere [1, 35] and confirmed by PCR. The expected phenotypes of all deletion mutants used in the study have been confirmed in former experiments with cell lines.

### Gentamicin protection assays

The medium of the cultured cells was changed to DMEM without P/S and supplemented with 10% FCS and recombinant M-CSF (25 ng/mL) at least 12 h before infection. Cells were starved in DMEM medium without FCS for 1 h before inoculation. Overnight cultures of bacteria were added to the primary cells at a multiplicity of infection (MOI) of 5:1 and plates were centrifuged at 250 × g for 3 min to synchronize infection.

After 30 min of incubation, the cells were washed with phosphate-buffered saline (PBS) and then maintained in infection medium (DMEM medium supplemented with 10% FCS and 50 µg/ml gentamicin (Sigma-Aldrich)) to eliminate extracellular bacteria. This approach enables the investigation of the intracellular replication dynamics of *Lm* in primary bovine microglia and MDM through colony-forming unit (CFU) quantification. For quantification of intracellular bacteria, cells were washed twice with PBS and then lysed with 0.5% ice-cold TritonX-100 (Sigma-Aldrich) at 45 min, 2 h, 4 h, 6 h, and 24 h post infection (pi) depending on the experiment. Multiple dilutions (1:1, 1:10, 1:100, 1:1′000, 1:10′000) were plated on BHI plates for CFU quantification. The resulting CFU counts were normalized to the inoculum. At least five independent experiments with cells from individual animals (five biological replicates) were performed in technical triplicates.

### Immunofluorescence (IF) of primary cell cultures

Cells were infected in the gentamicin protection assay as described above on glass coverslips or in 96-well optical plates (IBIDI®, GMBH). LysoTracker Red DND-99 (L7528, ThermoFisher Scientific) staining was performed according to the manufacturer’s instructions. Briefly, cells were incubated with infection medium containing 50 nM LysoTracker 30 min before the desired time point. For all markers, cells were washed with PBS at the indicated time points, fixed with 4% paraformaldehyde (PFA) for 30 min at 37 °C and stored in PBS at 4 °C until further analysis. Prior to immunofluorescence (IF) labeling, cells were washed with PBS-0.1% Tween (PBS-T) and incubated in 0.5% Triton-X100 in PBS for five minutes. Cells were then incubated with primary antibodies in PBS-T containing 10% normal goat serum (NGS) for one hour in the dark at room temperature. Primary antibodies included anti-Lamp1 (ab24170, abcam; 1:100), LLO (abcam; 1:100), and LC3b (PA1-46286, ThermoFisher Scientific; 1:100). Following incubation with the primary antibodies, cells were carefully washed three times with PBS-T and incubated with the secondary antibody (Life Technologies AlexaFluor® 555 goat anti-rabbit IgG, diluted at 1:500 in PBS-T with 10% NGS), DAPI (1:10,000), and phalloidin DyLight 633 (Invitrogen; 1:500) for one hour in the dark at room temperature. The cells were then washed three times with PBS-T. Cells in optical wells were kept in PBS-T at 4 °C until imaging. Coverslips were rinsed with H_2_O, mounted with Glycergel (Dako, Baar, Switzerland), and stored at 4 °C overnight.

### Immunofluorescence image acquisition and analysis

Immunofluorescence images of brain tissues from neurolisteriosis cases and infected primary cell cultures were acquired using an Olympus FV3000 confocal laser scanning microscope and analyzed with Fiji (ImageJ) [69].

In tissue sections, a total of 52 microabscesses were imaged in Z stacks at 60 × magnification. Semi-automated cell quantification was performed using Fiji [69]. Due to variability in microabscesses size, cell counts for each marker were normalized to the total number of nuclei per microabscess. Nuclei were identified and quantified using the StarDist2D plugin (https://imagej.github.io/imagej-wiki-static/StarDist.html). The association of *Lm* with microglia and MDM was assessed through both manual counting and object-based colocalization analysis in Fiji, using the BIOP-JACoP plugin (https://github.com/BIOP/ijp-jacop-b?tab=readme-ov-file). Briefly, following denoising and background subtraction, images were processed in BIOP-JACoP, and colocalization was quantified using the M1 and M2 coefficients derived from thresholded images (MaxEntropy or Otsu). The area of overlap between *Lm* and microglia (P2RY12) or MDM (F13A1) was quantified based on fluorescence signal co-localisation.

In infected primary cell cultures, manual quantification of host cells, bacterial number, and marker association was performed using the Cell Counter plugin in Fiji [69]. One author, blinded to experimental conditions (e.g., whether the sample was microglia or MDM), manually counted all host cell nuclei, individual bacteria, and positive marker signals per image. For each time point and condition, a minimum of 10 representative fields per well were analyzed. In total, 49′823 bacteria were counted to determine the average number of bacteria per cell and the percentage of actin-polymerizing bacteria. Additional details on the number of bacterial quantification and marker association are provided in SI Appendix, Table S1.

### Phagosome protection assay

Infection was performed in a gentamicin protection assay. The phagosome protection assay was performed according to a previously described protocol [50]. Briefly, at each time point, cells were washed with KHM buffer (110 mM potassium acetate, 20 mM HEPES, 2 mM MgCl_2_, pH 7.3) and permeabilized for 1 min with 50 µg/ml digitonin (Cat# D5628, Sigma-Aldrich) in KHM buffer. After 1 min, the cells were washed with KHM buffer, and incubated with rabbit Listeria O antiserum (1:200) in KHM supplemented with 3% bovine serum albumin (BSA, Cat# A2153, Sigma-Aldrich) for 15 min at 37 °C in 5% CO_2_. Cells were washed with PBS, fixed with 4% PFA for 15 min, washed with PBS and then incubated with the secondary antibody (AlexaFluor® 555 goat anti-rabbit IgG; Life Technologies; 1:500), DAPI (1:10,000) and phalloidin DyLight 633 (Invitrogen; 1:500) for 1 h at room temperature. Cells were then treated for IF as described in the previous section.

### Transmission electron microscopy

*Lm*-infected (MOI = 5) primary bovine microglia and MDM were processed for TEM at 6 h and 24 h post infection, as previously described [53]. Briefly, cells were washed with 100 mM sodium cacodylate buffer (pH 7.3) and fixed with cacodylate buffer containing 2.5% glutaraldehyde for 10 min. Cells were collected using a rubber cell scraper and centrifuged at 1000* g* for 10 min at room temperature. The supernatant was removed, and the cells were further fixed in glutaraldehyde-cacodylate overnight at 4 °C. Postfixation was performed in 2% OsO4 in cacodylate buffer for 2 h at room temperature, followed by extensive washing in water. After prestaining in saturated uranyl acetate (Cat-N°22409, Electron Microscopy Sciences, Hatfield) for 1 h at room temperature, specimens were dehydrated in a graded series of ethanol (30%, 50%, 70%, 90%, 3 times 100%) and embedded in Epon 820 epoxy resin, with two resin changes during 48 h. Polymerization of the resin was achieved by incubation at 65 °C for 24 h. Ultrathin sections of 80 to 90 nm were cut on a Reichert and Jung ultramicrotome. Sections were mounted on 300-mesh copper grids (Plano GmbH), and samples were stained with uranyl acetate (Cat. N°22409, Electron Microscopy Sciences, Hatfield) and lead citrate (Cat. N°22410, Electron Microscopy Sciences, Hatfield). Samples were examined on a FEI Morgagni Transmission Electron Microscope. One hundred infected cells were analyzed at 6 h and 24 h post infection for the presence, morphological integrity and subcellular location of bacteria.

### LIVE/DEAD™ BacLight bacterial viability

To assess the viability of intracellular *Lm*, we used the Live/Dead BacLight™ bacterial viability kit (ThermoFischer Scientific, L7012) following the manufacturer’s instructions with slight modifications as previously described [5, 38]. This assay utilizes two fluorescent nucleic acid dyes, SYTO9 and propidium iodide (PI), to differentiate live from dead bacteria based on membrane integrity. SYTO9, a green-fluorescent dye, penetrates and stains all bacteria, regardless of their viability, while PI, a red-fluorescent dye, selectively penetrates bacteria with compromised membranes, indicating bacterial death. Bacteria displaying SYTO9⁺/PI⁻ (green) staining were classified as viable, while PI⁺ or SYTO9⁺/PI⁺ (red or orange) bacteria were considered non-viable. Quantification was performed by analyzing fluorescence profiles in infected cells, with bacterial viability expressed as the proportion of SYTO9⁺/PI⁻ bacteria relative to the total bacterial signal. To estimate the number of viable but non-culturable (VBNC) bacteria, results from the BacLight™ assay were compared to colony-forming unit (CFU) counts. Both CFU and BacLight™ data were normalized to the number of host cells, and the VBNC population was calculated as the difference between viable bacteria per host cell (SYTO9⁺/PI⁻) and CFU per host cell. All experiments were performed in at least 3 independent biological replicates, with BacLight™ staining and CFU counting conducted in parallel.

### Statistical analysis

Statistical analyses were performed using Prism 9 (GraphPad Software Inc.). Comparisons of numerical outcomes between two groups were assessed using the non-parametric Mann–Whitney U-test (unpaired, two-tailed). When results are shown in percentages (Figs. 3, 5 and 6), the statistical analysis were performed on the absolute numbers (bacteria associated with a specific marker per cell) to assess the significance of differences between conditions. The level of significance was set to 0.05 where * p < 0.05; ** p < 0.01; *** p < 0.001 and **** p < 0.0001. Non-significant differences (or lack of evidence of differences) are indicated in the figures as “ns”.

## Results

### *Lm* targets microglia in early neurolisteriosis lesions

Previous work from our group has demonstrated the efficient spread of *Lm* within the brain, while being closely chased by phagocytes [35], ultimately leading to the formation of microabscesses with high numbers of *Lm* during early neurolisteriosis [35, 55]. Furthermore, we previously showed that *Lm* preferentially targets microglia in an organotypic brain slice model [32]. Building on these findings, the present study systematically analysed microabscesses from naturally occurring bovine neurolisteriosis cases to examine the interactions between *Lm*, microglia, and MDM.

To this end, we analysed microabscesses at different stages with immunofluorescence (IF) using antibodies against P2RY12 and F13A1, which we have previously validated as specific markers for bovine microglia and infiltrating MDM, respectively [76]. A total of 52 microabscesses were categorised into three stages based on their cell type composition, as described in Material and Methods: type I (early, n = 5), type II (acute, n = 12) and type III (subacute to chronic, n = 35) (Fig. 1a). In agreement with our earlier observations that microabscesses progressively spread from the brainstem to more rostral brain regions such as the midbrain [35, 55], early-stage microabscesses were mainly located in the midbrain, while more advanced microabscesses were found predominantly caudally in the medulla oblongata. Type I microabscesses were associated with inconspicuous to thin perivascular cuffs composed of lymphocytes and MDM (SI Appendix, Fig. S1). In contrast, type II and III microabscesses were surrounded by more prominent, multilayered perivascular cuffs containing numerous lymphocytes, MDMs, occasional neutrophils, and rare plasma cells. Additional histopathological changes—including axonal degeneration, edema and astrocytic gliosis – were variably present.

In early microabscesses, microglia outnumbered MDM with an average of 26% P2RY12^+^ microglia versus 9% F13A1^+^ MDM of all nucleated cells (p value < 0.05) (Fig. 1b–d). In acute microabscesses, the fraction of MDM increased to 29%, while the P2RY12^+^ microglia fraction declined significantly to 6% (p value < 0.0001). At this stage, microglia and MDM were frequently observed surrounding a large central core of neutrophils, as identified by nuclear morphology (Fig. 1a, b, c, SI Appendix, Fig. S2). Subacute to chronic microabscesses showed the highest proportion of MDM (37%) with scattered residual microglia (11%, p value < 0.0001) localized within or at the margins of lesions (Fig. 1b–d, SI Appendix, Fig. S2).

*Lm* were most abundant in early microabscesses and showed a strong association with microglia: 28% of P2RY12^+^ microglia contained *Lm*, compared to only 3% of F13A1^+^IBA-1^+^ MDM (p value < 0.05) (Fig. 1b–d). Microglia also more frequently contained larger numbers of bacteria, as confirmed by quantification of the mean area of overlap between *Lm* and P2RY12 versus F13A1, and by Mander’s colocalization coefficients (SI Appendix, Fig. S2). These findings suggest that *Lm* replicates within the brain-resident macrophage population during the early phase of infection. In acute microabscesses, the proportion of *Lm*-infected cells declined in both cell populations: 6% of P2RY12^+^ microglia and 1% of F13A1^+^IBA-1^+^ MDM contained *Lm* (Fig. 1d). In subacute to chronic microabscesses, this dropped further to 0.65% in microglia and 0.35% in MDM. At this stage, both infected macrophage types typically contained only single bacteria (SI Appendix, Fig. S2). Collectively, these findings show that during the early stages of neurolisteriosis, microglia are the primary host cells harboring the highest *Lm* burden among macrophage subtypes, suggesting *Lm* replication within microglia but not within MDM during the initial phase of CNS infection.

### *Listeria monocytogenes* efficiently replicates within the cytosol of cultured primary microglia, but not in MDM

**Fig. 2:**
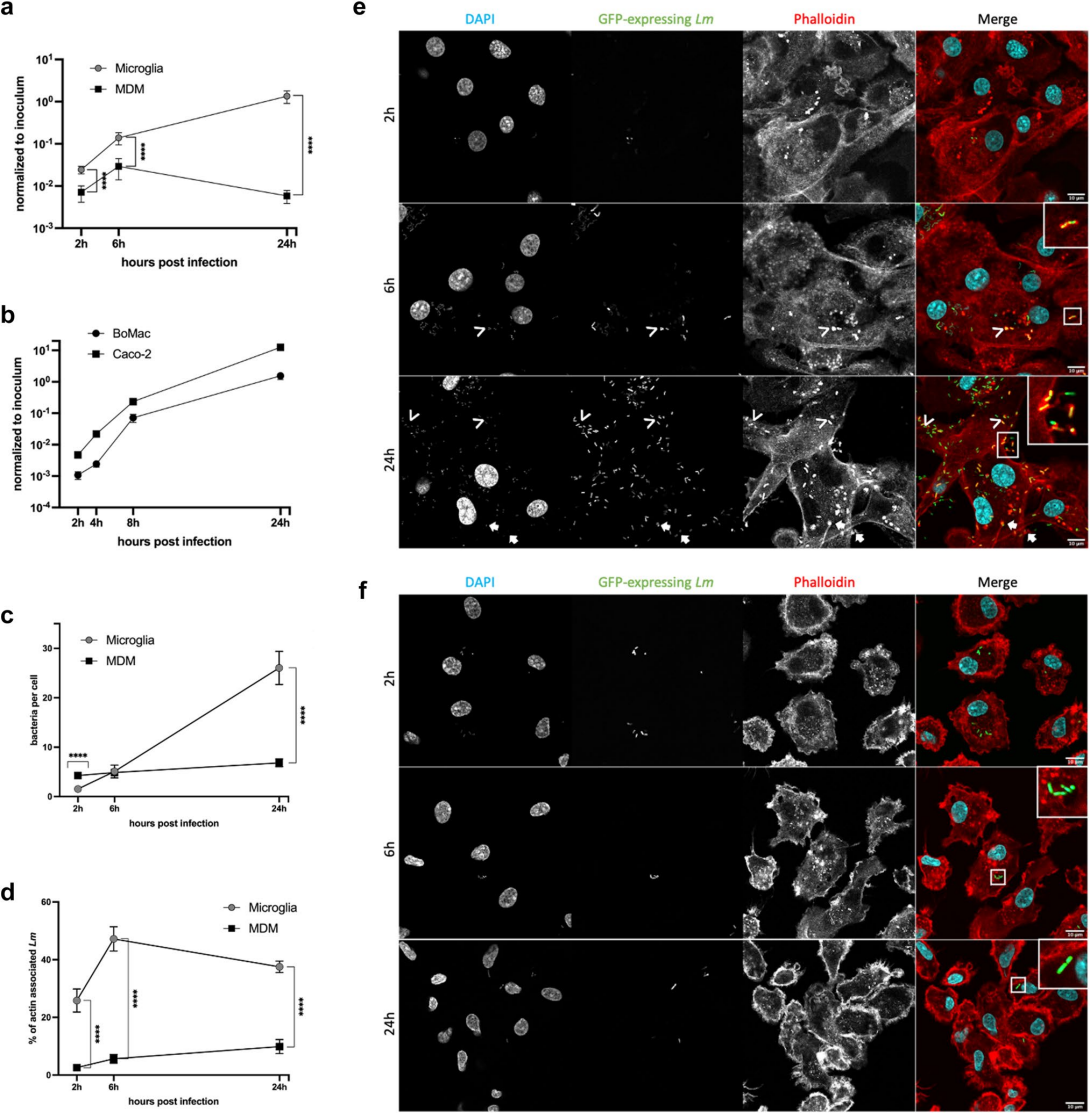
While in microglia *Lm* proliferates within the cytosol and spreads from cell to cell using the actin machinery, MDM maintain *Lm* number at bay. Intracellular behaviour of *Lm* in microglia and MDM assessed using a gentamicin protection assay and quantified with colony forming units (CFU) and immunofluorescence (IF) at indicated time points. **a** While MDM control *Lm* infection, microglia support the intracellular *Lm* growth, with bacterial numbers exceeding the inoculum at 24 h post infection (pi) by nearly two orders of magnitude. Data from 12 independent experiments in biological replicates (primary cells from individual animals) per cell type, performed in triplicate, with CFU normalized to the inoculum. **b** Intracellular CFU dynamics in microglia resemble infection in non-phagocytic cells (Caco-2 and BoMac cell lines, data from Gözel et al., 2019 and Rupp et al., 2017). Manual quantification (using the CellCounter plugin in Fiji) of intracellular bacteria using IF images: **c** While in microglia the number of bacteria per cell sharply increases from 2 to 24 h, in MDM the number remains stable over all time points; **d** Percentage of *Lm* with polymerized actin (actin clouds and tails). A significantly higher proportion of bacteria in microglia is associated with polymerized actin than in MDM. Data shown in **c**, **d** are from 5 independent experiments in biological replicates (primary cells from individual animals) per cell type. A minimum of 10 images were acquired per time point, totalling 49,823 bacteria assessed (32,745 in microglia and 17′078 in MDM). Error bars indicate SEM, **** p < 0.0001 (Mann–Whitney U test). Representative IF images of microglia and MDM at 2 h, 6 h and 24 h post infection: in microglia (**e**), numerous *Lm* (green) are associated with polymerized actin (red, insets) in the form of actin clouds (arrowheads at 6 and 24 h) and actin tails (arrows 24 h), indicating intracytosolic localization; **f** In MDM, there are very few bacteria along the 24 h infection, with no visible actin polymerization (inset), indicating intravacuolar localization. In merged images, nuclei are stained cyan (DAPI), actin is stained red (phalloidin) and bacteria are stained green, with white squares indicating the location of the insets

Following our in situ observations, we used a gentamicin protection assay to investigate the intracellular lifestyle and replication dynamics of *Lm* in primary bovine microglia and MDM through colony-forming units (CFU) quantification and IF. We used a sequence type 1 (ST1) clonal complex 1 (CC1) *Lm* strain (JF5203) [67]. Wild type (JF5203-wt) *Lm* expressing green fluorescent protein (GFP) showed two distinct infection phenotypes in microglia and MDM cultured under the same conditions (Fig. 2a). Notably, after 2 h, higher CFU numbers were recovered from microglia compared to MDM (p value < 0.0001), with this discrepancy becoming more pronounced over time. By 24 h, CFU counts in microglia increased by approximately two orders of magnitude, mirroring the exponential intracellular growth observed in epithelial cell lines (Fig. 2b, p value < 0.0001) [29, 67]. In contrast, intracellular bacterial CFU in MDM showed minimal increase between 2 and 6 h and returned to baseline levels at 24 h. Our findings indicate that while *Lm* survives in both macrophage types, its fate differs significantly between microglia and MDM. Specifically, *Lm* replicates efficiently in microglia at rates comparable to those seen in non-phagocytic cells, whereas its replication in MDM is notably limited.

Because the differences in CFU recovered at 2 h suggested potential differences in bacterial internalization between the two macrophage types, we tested *Lm* mutants of JF5203-wt with deletions of the genes *inlA* (internalin A) and *inlB* (internalin B), which encode major bacterial surface invasion proteins. JF5203-Δ*inlA* and JF5203-Δ*inlB* were not impaired in invasion of either MDM or microglia compared to JF5203-wt (SI Appendix, Fig. S3). As in the parental strain, higher CFU were recovered from microglia than from MDM infected with the deletion mutants. These data confirm that *Lm* do not invade bovine MDM or microglia by actively triggering internalization through the interaction with host receptors including E-cadherin and c-met [10, 44], but rather are taken up by phagocytosis. However, data from a previous study provided evidence that MDM are more efficient than microglia at phagocytosing particles [76], and live-cell imaging showed that bovine MDM phagocytose *Lm* more rapidly and efficiently (SI Appendix, Video S1). Consequently, we aimed to further elucidate the intracellular lifecycle of *Lm* in these two macrophage populations by microscopy.

IF quantification of bacterial numbers per microglial and MDM cell in 5 independent experiments confirmed significant discrepancies in *Lm* intracellular dynamics between the two macrophage populations observed by CFU quantification (Fig. 2c). However, in contrast to the CFU quantification, bacterial counts per cell quantified by IF imaging were slightly but significantly higher in MDM than in microglia at the 2 h time point (p value < 0.0001, Fig. 2c; SI Appendix, Fig. S4). Taken together, these data indicate that the CFU readout underestimates intracellular bacteria in MDM. While intracellular bacterial counts in microglia strongly increased over 24 h (Fig. 2c, e), bacterial numbers in MDM remained at a constant low level throughout the infection (Fig. 2c, f, p value < 0.0001).

Quantification of nearly 50,000 GFP-expressing *Lm* (Fig. 2d, SI Appendix, Table S1) showed that between 20 and 50% of bacteria in microglia polymerized actin in the form of actin tails and clouds (Fig. 2e, SI Appendix, Fig. S4), an indicator of cytosolic location [42], whereas the proportion of actin-polymerizing bacteria in MDM remained consistently low (maximum 15%, p value < 0.0001). These data suggest that *Lm* escape efficiently into the cytosol of microglia, where they replicate, whereas in MDM, the large majority of *Lm* are confined to vacuolar compartments preventing their rapid intracytosolic replication (Fig. 2d, f). To confirm the differences in intracellular bacterial localization between microglia and MDM, we quantified cytosolic and vacuolar bacteria using the phagosome protection assay (SI Appendix, Fig. S4 and Fig. S5). This method exploits the ability of the cholesterol-binding detergent digitonin to selectively permeabilize the plasma membrane, while leaving intracellular membranes intact, allowing selective staining of cytosolic bacteria with antibodies [50]. Throughout the infection experiment, we observed a higher percentage of cytosolic bacteria in microglia (over 20%) than in MDM (less than 10%) (SI Appendix, Fig. S2f and SI Appendix, Fig. S3, p value < 0.0001). Although the phagosome protection assay underestimated the cytosolic location of bacteria compared to the actin staining, likely due to interference of the polymerized actin with the *Lm* antibody labelling (SI Appendix, Fig. 5), it confirmed the difference in the proportions of intravacuolar versus intracytosolic bacteria between microglia and MDM.

**Fig. 3:**
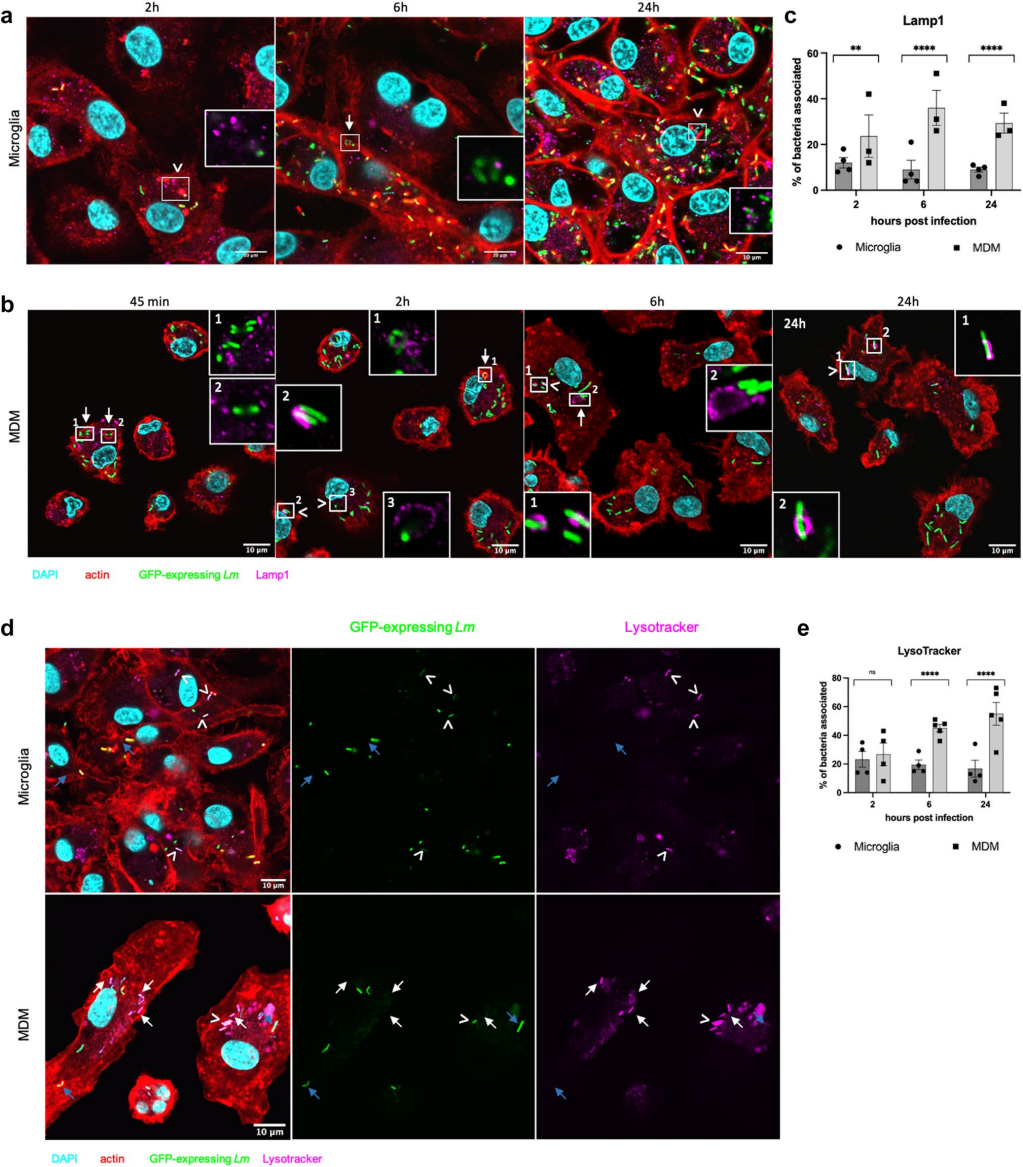
Association of *Lm* with acidic vacuoles is more pronounced in MDM compared to microglia. Representative images of *Lm* (green) association with Lamp1 (pink) structures with red-labelled actin **a** in microglia at 2 h, 6 h, and 24 h post infection (pi) and **b** MDM, at 45 min, 2 h, 6 h, and 24 h pi. Two types of Lamp1-expressing structures were observed more frequently in MDM: (1) large vacuolar structures, often containing several bacteria, resembling SLAPs (white arrows; higher magnifications in white-framed insets); and (2) small vacuoles tightly surrounding single bacteria (white arrowheads; higher magnifications in white-framed insets). **c** Quantification (using the CellCounter plugin in Fiji) of Lamp1-associated bacteria reveals a significantly higher association with Lamp1 in MDM than in microglia. **d** Representative images of *Lm* (green) association with LysoTracker (pink) in microglia and MDM at 6 h pi. White arrowheads indicate LysoTracker-associated bacteria with quenched GFP-signal, white arrows indicate Lysotracker-associated bacteria with lost GFP-signal, and blue arrows indicate GFP-expressing bacteria not associated with LysoTracker. **e** Quantification of LysoTracker-associated bacteria (using the CellCounter plugin in Fiji) at 2, 6 and 24 h pi demonstrates that the proportion of LysoTracker-associated bacteria increases over time in MDM but not in microglia. Cellular and bacterial nuclei are stained in cyan (DAPI), GFP expressing JF5203-wt *Lm* (green), cellular actin with phalloidin (red). Each data point in **d**, **e** represents an individual experiment, using primary cells obtained from individual animals for each cell type. A minimum of 10 images were acquired per time point per experiment, totalling 8′827 bacteria in **d** and 10′812 bacteria in **e**. At least three independent experiments were performed. Error bars indicate SEM, ns: not significant, ** p < 0.01, and **** p < 0.0001 (Mann–Whitney U test performed in absolute numbers (see SI Appendix, Fig. S4))

Taken together, these results indicate that microglia allow *Lm* to escape from the phagocytic vacuoles prior to phagolysosomal fusion and to replicate in the intracytosolic niche with similar efficiency as in non-phagocytic cells (Fig. 2b). In contrast, in MDM, *Lm* appears to be forced into a predominantly vacuolar lifestyle, in which most bacteria are trapped in phagosomes without reaching the cytosol, preventing their rapid intracellular replication. However, MDM are unable to completely eliminate bacteria.

### Maturation dynamics of bacteria-containing phagosomes differ between cultured primary microglia and MDM

In line with existing literature [17, 46, 79], our in vitro findings indicate that phagosomal retention of bacteria within MDM is an essential mechanism for controlling infection, whereas this mechanism does not appear to be activated in microglia cultured under identical conditions, consistent with our observations in early brain lesions. Therefore, we sought to characterize the phenotype of bacteria-containing compartments using a set of different host markers and transmission electron microscopy (TEM).

Lamp1 (lysosome-associating membrane protein-1) is a late phagosomal and phagolysosomal marker, crucial for maintaining phagolysosomal integrity [2, 23]. Recent studies have identified Lamp1 decoration on vacuolar niches harbouring *Lm* including SLAP, eSLAP, and LisCV structures [11, 41, 59]. Consistent with our actin polymerization data, the proportion of Lamp1-associated *Lm* (Fig. 3a–c, SI Appendix, Fig. S4) differed between microglia and MDM. In microglia, the fraction of *Listeria* associated with Lamp1 remained close or below 10% over the 24 h course of infection. In contrast, in MDM, bacterial Lamp1 association consistently exceeded 20% throughout the 24 h (p value < 0.0001). Additionally, actin-lined large vacuolar structures containing multiple Lamp1-associated bacteria, resembling SLAPs, were observed exclusively in MDM, but not in microglia (Fig. 3b). These observations suggest that 1) a subset of intraphagosomal bacteria associates with Lamp1-decorated phagosomes in both macrophage populations, and 2) bacteria in MDM are retained in Lamp1 positive and negative phagosomes.

LC3b, a marker related to LC3b associated phagocytosis (LAP pathway), a form of non-canonical autophagy, has been shown to contribute to *Lm* killing [28]. Interestingly, while the proportion of LC3b-associated bacteria did not exceed 15% in either microglia or MDM throughout the 24 h infection, intracellular bacteria were more frequently associated with LC3b in MDM, with the proportion increasing during the infection experiment (SI Appendix, Fig. S4, SI Appendix, Fig. S6, p value < 0.001). These data suggest that although the LAP pathway is more activated in MDM than in microglia, MDM predominantly restrict *Lm* to LAP-independent vacuoles, with only a small fraction of bacteria entering the LAP pathway.

Infection assays using LysoTracker, a cell-permeable fluorescent dye that stains acidic cell compartments including late phagosomes, lysosomes and phagolysosomes [40, 45], further confirmed differences in the phenotype of bacteria-containing vacuoles between microglia and MDM (p value < 0.0001). Whereas no more than 23% of *Lm* were located in acidic vacuoles of infected microglia over 24 h of infection (Fig. 3d, e, SI Appendix, Fig. S4), the proportion of bacteria contained in acidic vacuoles in MDM increased from 26% at 2 h to over 55% at 24 h (Fig. 3d, e, SI Appendix, Fig. S4). Bacterial association with LysoTracker coincided with a quenched bacterial GFP signal, more frequently observed in MDM, further supporting their frequent localization within acidic compartments (Fig. 3d). To confirm that GFP-quenching was related to acidic pH rather than bacterial death, we assessed the pH-sensitivity of our GFP expressing *Lm* strain (JF5203-wt) (SI Appendix, Fig. S7). Indeed, the strain exhibited almost complete loss of GFP signal after exposure to pH5 for 24 h, which was restored upon return to neutral pH conditions (SI Appendix, Fig. S7).

**Fig. 4:**
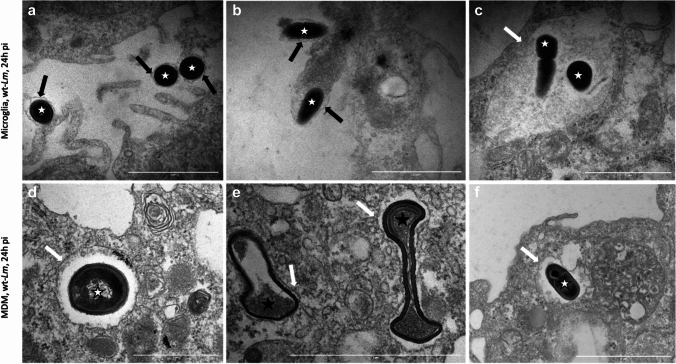
Monocyte-derived macrophages (MDM) and microglia exhibit bactericidal activity against *Lm*. Representative transmission electron microscopy images of JF5203-wt *Lm* in MDM (upper panel) and microglia (lower panel) infected at 24 h post infection (pi) illustrating intact bacteria (white stars in **a–d**, **f**) or disintegrated bacteria (black stars in **e**) within single membrane vacuoles (white arrows in **c–f**) or in the cytosol of microglial filopodia (“listeriopods”, **a**, **b**). Black arrows in Panels **a** and **b** indicate the plasma membrane surrounding bacteria

TEM analysis of infected microglia and MDM at 6 h and 24 h (Fig. 4) post infection revealed that *Lm* resided in single membrane-bound vacuoles in both cell types throughout the infection, indicating that the majority of intravacuolar bacteria had entered a phagocytic (either conventional phagocytosis and/or LAP), but not autophagic pathway [28]. In addition, large vacuoles containing multiple bacteria, together with moderately electron dense material, as well as small vacuoles, containing single bacteria enclosed by tight membranes, were found in both microglia and MDM (Fig. 4c-f). Furthermore, *Lm* was observed in the cytosol of microglia and, to a lesser extent, MDM. In microglia, but not in MDM, the presence of *Lm* with or without a discernible actin tail in the cytosol of filopodia (so-called listeriopods) [26] became apparent at 24 h (Fig. 4a, b). While the majority of intracytosolic and intravacuolar bacteria were morphologically intact in both microglia and MDM, we surprisingly observed intravacuolar bacteria with collapsed and disintegrated cell wall and protoplasm in both cell types (Fig. 4e). These results reveal that not only MDM but also microglia are able to kill a proportion of *Lm*.

Taken together, our data suggest that phagosomal maturation dynamics differ between infected microglia and MDM, with MDM trapping *Lm* in heterogenous vacuolar compartments of phagocytic pathways, including a large proportion of single membrane-bound acidic compartments that may be either late phagosomes or phagolysomes. In addition to the acidic compartments, during early infection, *Lm* are trapped in large vacuolar structures resembling SLAPs (Fig. 3b) in MDM, pointing towards the ability of *Lm* to survive and possibly replicate intra-vacuolarly. Importantly, we observed that microglia are also capable of killing a subpopulation of intravacuolar bacteria, albeit tempered by the rapid replication of bacteria that have escaped into the cytosol.

### Deletion of bacterial *hly* licenses microglia to check intracellular bacteria

**Fig. 5:**
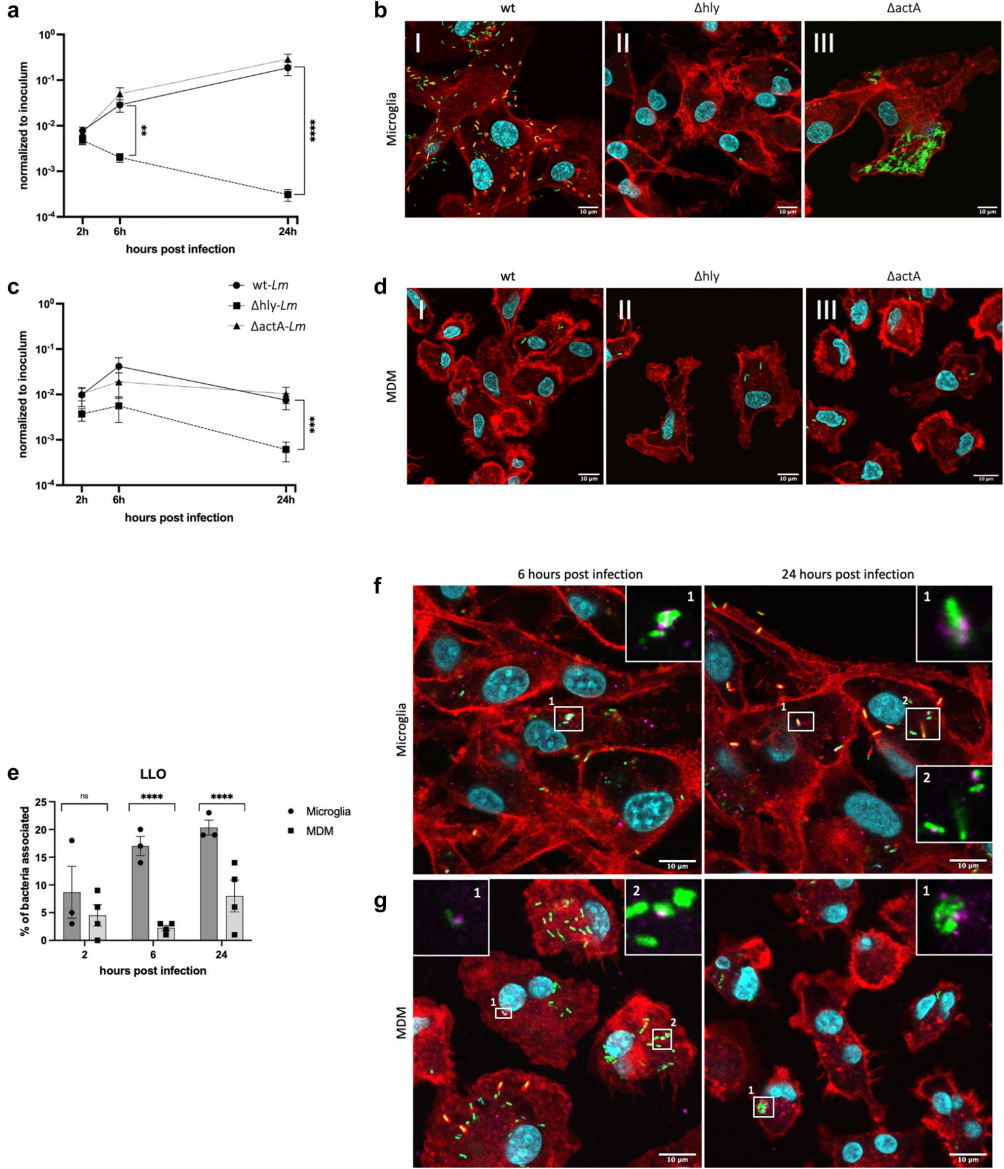
The infection of microglia with *hly* deletion mutant mirrors the phenotype of wt *Lm in* MDM. **a** CFU quantification of intracellular bacteria in microglia at 2 h, 6 h and 24 h post infection (pi) shows a marked CFU decrease in JF5203-Δ*hly* compared to the parental strain*,* while JF5203-Δ*actA* shows similar levels to the parental strain. **b** Representative immunofluorescence (IF) images at 24 h pi showing phenotypic differences between (I) JF5203-*wt*, (II) JF5203-Δ*hly* and (III) JF5203-Δ*actA*. While JF5203-Δ*hly* is clearly reduced in number compared to the parental strain, JF5203-Δ*actA* shows defective intercellular spreading with isolated microglia crowded with numerous bacteria. Both mutants lack actin polymerization in microglia. GFP-expressing bacteria are shown in green, DAPI in cyan, and phalloidin in red. **c** CFU quantification of intracellular bacteria in MDM at 2 h, 6 h and 24 h pi, revealing attenuation of JF5203-Δ*hly* that is less pronounced than in microglia, while JF5203-Δ*actA* behaves similarly to the parental strain. CFU data were obtained from six independent experiments using primary microglia and eight using primary MDMs, each derived from individual animals and performed in triplicates. Datapoints colour-coded by animal are shown in SI Appendix, Fig. S8. **d** Representative IF images at 24 h pi in MDM demonstrate a lack of clear differences in the infection phenotype between the parental strain (I) and the deletion mutants (II and III). **e** Quantification of bacteria expressing LLO (using the CellCounter plugin in Fiji) at 2 h, 6 h and 24 h pi in microglia (circles) and MDM (squares). The fraction of LLO expressing bacteria increases in MDM over time and is significantly higher than in microglia. A minimum of 10 images were acquired per time point, per experiment resulting in the quantification of 9′536 bacteria. Six and eight independent experiments were performed for JF5203-Δ*hly* and JF5203-Δ*actA* in microglia (**a**) and in MDM (**c**), respectively. Representative images of LLO (pink) colocalization with GFP expressing JF5203-wt *Lm* (green) in microglia (**f**) and MDM (**g**) at 6 h and 24 h pi. Cellular and bacterial nuclei are stained in cyan (DAPI) and actin in red (phalloidin). White squares indicate the location of the insets with higher magnification. Error bars indicate SEM, ns: not significant, ** p < 0.01, *** p < 0.001 and **** p < 0.0001 (Mann–Whitney U test performed in absolute numbers (see SI Appendix, Figure S4))

Listeriolysin O (LLO) and ActA are recognized as crucial virulence factors in the intracellular life cycle of *Lm*. To explore their involvement in bacterial fate and vacuolar lifestyle within MDM and microglia, we infected both cell types with JF5203-derived *Lm* mutants containing deletions of *hly* and *actA*, genes encoding LLO and ActA, respectively. JF5203-Δ*hly* is unable to escape the vacuole [27], while JF5203-Δ*actA* can escape the vacuole and replicate in the cytosol but is severely impaired in spreading to neighbouring cells [35, 67]. In microglia, the intracellular CFU counts of JF5203-Δ*hly* decreased rapidly, diminishing by more than one order of magnitude at 24 h. This reduction resulted in a difference of more than two orders of magnitude in CFU numbers between JF5203-wt and JF5203-Δ*hly* in all independent experiments at 24 h (Fig. 5a and SI Appendix, Fig. S8A, p value < 0.0001), mirroring the CFU kinetics of JF5203-wt in MDM. Surprisingly, *hly* deletion also resulted in a significant reduction of CFUs in MDM, contrary to expectations given its predominantly intravacuolar location (p value < 0.001). Although consistently observed across all independent experiments, this effect was less pronounced than in microglia (Fig. 5c and SI Appendix, Fig. S8B). Noteworthy, in the absence of LLO expression, the number of intracellular bacteria dropped below the initial count in both microglia and MDM, suggesting that in addition to mediating vacuolar escape, LLO may also contribute to intravacuolar survival [11].

IF showed a significant reduction in the number of intramicroglial JF5203-Δ*hly* compared to JF5203-wt, consistent with CFU data, and a complete absence of actin polymerization (Fig. 5b, SI Appendix, Fig. S9), mimicking the phenotype of JF5203-wt in MDM. These findings provide further evidence that in the absence of LLO, *Lm* fails to access the cytosol of microglia for replication and spread. In contrast, the microscopic infection phenotype of JF5203-Δ*hly* in MDM closely resembled that of the JF5203-wt strain, with both exhibiting low bacterial numbers and minimal to no actin polymerization (Fig. 5d, SI Appendix, Fig. S4). These findings further support our previous observations indicating that *Lm* resides within intravacuolar compartments in MDM. Hence, we speculated that in MDM, bacterial LLO expression of JF5203-wt is insufficient to trigger vacuolar escape. IF analysis showed that *Lm* expressing LLO were very seldom surrounded by actin, suggesting that LLO expression occurs in the intravacuolar stage of *Lm*, consistent with its role in vacuolar escape (Fig. 5f, g) [59]. During microglia infection, the proportion of intracellular bacteria expressing LLO increased to over 20% of the total bacterial population, whereas in MDM, the proportion of LLO-expressing bacteria remained consistently lower, reaching only 12.5% at 24 h (Fig. 5e, p value < 0.0001). LLO expression in MDM was observed not only in single intravacuolar bacteria but also in SLAP-like structures containing several bacteria (Fig. 5g) [11, 74, 77]. Similarly, deletion of *actA* had a notable effect on the infection phenotype in microglia but not in MDM (Fig. 5a–d). The effect was evident in IF-analysis (Fig. 5b, SI Appendix, Fig. S9), which showed a complete absence of actin-polymerization and the presence of numerous scattered infected microglia 24 h post infection, packed with bacteria due to intracytosolic replication of spread-defective bacteria. This focal replication of spread-defective bacteria most likely accounted for the lack of reduction in CFU numbers compared to the wild type (Fig. 5a), as it has been described in other host cell types [35, 67]. In MDM, deletion of *actA* did not result in any decrease in intracellular CFU numbers (Fig. 5c). IF revealed no actin tails in MDM (Fig. 5d, SI Appendix, Fig. S9), and cells packed with bacteria were only infrequently observed.

Collectively, these data provide further evidence that *Lm* replication in microglia occurs in the cytosol and requires escape from the phagosome prior to phagolysosomal fusion. In MDM however, intracellular bacteria are mainly prevented from replicating by entrapment in late phagosomes or phagolysosomes, where the number of LLO-expressing bacteria is low.

### *Listeria monocytogenes* hides in MDM phagosomes as viable but non-culturable (VBNC) bacteria and lack of LLO induces VBNC in microglia

**Fig. 6:**
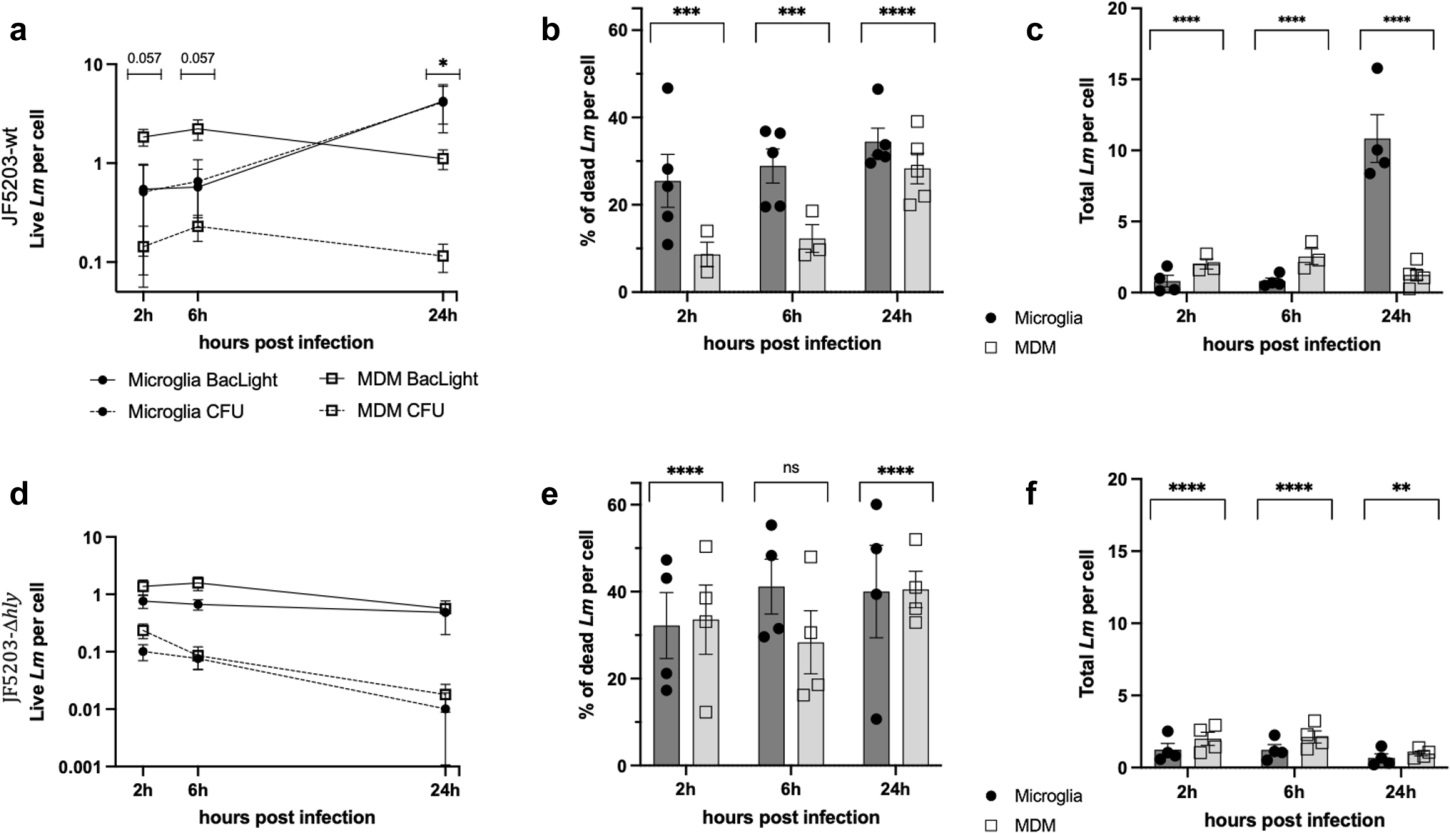
Microglia and monocyte-derived macrophages (MDM) exhibit comparable killing rates against *Listeria monocytogenes (Lm)*, but MDM induce viable but non-culturable *Lm*. BacLight™ assay to differentiate live from dead JF5203-wildtype *Lm* (upper panel, **a–c**) and JF5203-Δ*hly* (lower panel, **d–f**) in microglia and MDM based on bacterial membrane integrity. **a–f** Comparison between intracellular CFU and SYTO9-stained live bacteria, quantified in parallel in three experiments, and normalized to the number of host cells. Black solid circles indicate bacteria in microglia and empty squares indicate bacteria in MDM quantified either microscopically in BacLight™ (solid line) or by CFU (dashed line). **b**, **e** Percentage of dead bacteria per cell and **c**, **f** total bacteria number per cell as quantified in the BacLight™ assay. Compared to the number of Syto9-positive bacteria CFU counts underestimate the number of viable intracellular JF5203-wt in MDM (**a**), suggesting that nearly 90% of intravacuolar *Lm* in MDM are VBNC bacteria that are not detected by bacterial culture. In contrast, VBNC bacteria are not induced in microglia. While microglia kill a higher percentage of bacteria (**b**), they also contain increasing numbers of intracellular bacteria compared to MDM (**c**). JF5203-Δ*hly* (**d**) shows a similar phenotype in both microglia and MDM, mimicking the parental strain in MDM (**a**) with respect to differences in readouts between CFU quantification and BacLight™ data. While the killing rate of JF5203-Δ*hly* increases only in MDM, reaching microglia levels (**e**), the total number of intracellular JF5203-Δ*hly* remains constant in both in microglia and MDM (**f**). These data indicate VBNC induction of JF5203-Δ*hly* but not of JF5203-wt in microglia. CFU of JF5203-Δ*hly* at 24 h drop below the CFU number of JF5203-wt in MDM, and JF5203-Δ*hly* is killed at higher rates by MDM than the parental strain, suggesting a role for *hly* in intravacuolar survival. Data are derived from at least three independent experiments. Error bars indicate SEM, ns: not significant, ** p < 0.01, *** p < 0.001 and **** p < 0.0001 (Mann–Whitney U test performed in absolute numbers (see SI Appendix, Fig. S10))

The presence of intact and disintegrated intravacuolar bacteria (Fig. 4) and the unexplained incongruence between CFU and microscopic bacterial enumeration at 2 h (Fig. 2a, c) prompted us to assess bacterial viability in both macrophage types using the BacLight™ assay. This microscopy-based technique distinguishes live (SYTO9^+^/PI^−^ bacteria) from dead bacteria (PI^+^ bacteria) based on membrane integrity. Analysis of the BacLight™ data confirmed our IF findings showing that MDM contain more bacteria than microglia at 2 h (Fig. 6), likely due to increased phagocytosis (SI Appendix, Video S1). Notably, CFU counts underestimated the number of viable intracellular JF5203-wt in MDM (Fig. 6a), suggesting that a substantial population (nearly 90%) of intravacuolar *Lm* in MDM are VBNC bacteria that are not detected by bacterial culture (Fig. 2a). In contrast, the number of SYTO9^+^/ PI^−^ live bacteria in microglia aligned with the CFU data, indicating that *Lm* does not convert to VBNC forms in microglia. Interestingly, although microglia exhibited a tendency for higher bacterial killing rates than MDM across time points, the absolute number of bacteria per cell in microglia significantly exceeded that in MDM at 24 h due to rapid intracytosolic replication (Fig. 6b, c, SI Appendix, Fig. S10). Taken together, these data provide evidence for the induction of VBNC bacteria in MDM but not microglia as early as 2 h post infection (Fig. 2a).

To confirm the negative impact of *hly*-deletion on intracellular survival, as indicated by CFU quantification, we performed the BacLight™ assay with JF5203-Δ*hly* infected microglia and MDM. Surprisingly, deletion of *hly* did not significantly increase the killing rate of microglia as suggested by CFU, but induced VBNC *Lm* in microglia, mirroring the infection phenotype of JF5203-wt in MDM (Fig. 6d–f, SI Appendix, Fig. S10). In contrast, in infected MDM, deletion of *hly* did not elevate the ratio of VBNC bacteria (Fig. 6d–f, SI Appendix, Fig. S10), but increased the bacterial killing rate at early time points (Fig. 6f).

This study unveils, that the majority of *Lm* in MDM survive as VBNC bacteria, rapidly induced by their containment in late phagosomes/phagolysosomes, while only a minority are killed. Similarly, microglia also eliminate a comparable fraction of intravacuolar *Lm*. However, in microglia, after efficiently escaping from phagosomes, *Lm* replicate within the cytosol, culminating in a productive infection. LLO emerges as a key determinant of *Lm’*s fate, dictating whether bacteria escape from the phagosome into the cytosol or remain confined within the phagosome, where they either die or persist as VBNC forms.

## Discussion

Microglia and MDM are central to the immune response in the CNS, particularly during bacterial neuroinfection. However, our understanding of their functions in this context remains incomplete. In this study, we unveil divergent intracellular fates of *Lm,* an intracellular bacterium causing fatal encephalitis, between resident microglia and infiltrating MDM. Our results show that *Lm* efficiently exploits the microglial cytosol for replication, thereby amplifying infection within the CNS. In contrast, MDM confine *Lm* within the phagolysosomal system, impeding replication and inducing a viable but non-culturable (VBNC) state in the hostile intravacuolar environment rather than efficiently eliminating *Lm*. Our findings introduce LLO as a contributing factor in determining *Lm*’s intracellular destiny, influencing whether the bacterium escapes to the cytosol or remains in a dormant VBNC state within vacuoles.

Previous studies of neurolisteriosis have highlighted that *Lm* spreads to and within the brain via neuroaxonal pathways [35]. However, bacterial amplification occurs in early phagocytic infiltrates (microabscesses) along the infected axons rather than during the neuroaxonal stage of infection [18, 35, 55]. In later stages of inflammation, when microabscesses are dominated by macrophages and infiltrated by T lymphocytes, the bacterial load decreases [18, 55], suggesting distinct roles for the phagocytes (microglia, neutrophils and MDM) involved in *Lm* infection. Our findings demonstrate that microglia act as bacterial amplifiers, increasing the bacterial load in early microabscesses, despite exhibiting slightly higher bacterial killing rates than MDM. They allow bacteria to escape from the vacuole into the cytosol, where exponential growth outpaces the microglial killing rate, leading to bacterial expansion. Supported by previous studies observing microglial targeting by *Lm* [31, 32], these results challenge the long-debated ability of microglia to actively combat infections, given their primary role in maintaining homeostasis [68, 75]. While they play a central role in safeguarding the CNS against infections, they also demonstrate permissiveness, enabling the proliferation of microorganisms such as *Cryptococcus neoformans* [51].

The mechanisms by which microglia themselves become infected, for example by probing infected neurons/axons, remain to be elucidated. Nonetheless, chemokine secretion by infected microglia is a key process in the recruitment of peripheral phagocytes to the intracerebral site of infection and in the control of CNS infections [21]. For instance, *Lm*-infected microglia induce chemotaxis of bovine neutrophils by secreting IL-8 and other chemokines [4, 22]. Similarly, during infection with the parasite *Toxoplasma gondii*, microglia produce the alarmin IL-1α, which is required to initiate immune cell infiltration and control parasite infection [9].

MDM, while less effective in bacterial killing compared to microglia, keep *Lm* in check by confining them within vacuoles, inhibiting bacterial replication. However, despite this containment, complete bacterial elimination is not achieved, as a substantial fraction of *Lm* rapidly transitions into VBNC bacteria within MDM, a phenomenon not observed in microglia. This behaviour allows *Lm* to remain viable under hostile intravacuolar conditions for at least 24 h and differs with other intracellular bacteria such as *Legionella* and *Salmonella*, which survive by creating replication niches within macrophage vacuoles [63].

The VBNC state, a form of persister state, serves as a survival strategy employed by various gram-positive and gram-negative bacteria to withstand hostile conditions by slowing down metabolic activity. This state impairs replication, preventing the formation of colonies. Recent research indicates a correlation between the dormancy level of persisters and the stress they encounter, with the VBNC state representing the extreme end of this spectrum [61]. However, under favourable conditions, VBNC bacteria can regain their ability to grow and hence may play a significant role in the adaptation, persistence, and transmission of *Lm* across various ecological niches [6, 48, 57]. While VBNC persisters are predominantly observed in hostile extracellular conditions, they have also been identified within the host for major bacterial pathogens such as *Staphylococcus aureus*, *Mycobacterium tuberculosis*, *Salmonella enterica*, and others [60].

Host-associated VBNC forms of *Lm* have only recently been detected in epithelial cells after 3 days of infection [41]. Our results show that *Lm* rapidly induces the VBNC state as strategy to persist within MDM and may, at least transiently, evade detection while spreading within the host. Interestingly, although VBNC *Lm* have not yet been formally demonstrated in vivo under natural conditions, the nonculturability of *Lm* in bovine listeriosis was reported as early as 1948, with reculturability after long-term storage at 4 °C [30].

This novel paradigm of phagocytes serving as a mobile niche for latent *Lm* carries significant implications for diagnosis, pathogenesis, epidemiology and treatment strategies. Hidden within MDM as VBNC bacteria, *Lm* may initiate new sites of infection leading to persistent and recurrent infections, while evading antibiotics and humoral immune mechanisms. It is noteworthy that asymptomatic carriage of *Lm* in the faeces of both humans and cattle is frequently reported, and has recently been associated with virulence, being more common in pathogenic *Listeria* strains than in non-pathogenic ones [33]. Whether asymptomatic carriage involves VBNC forms remains to be conclusively demonstrated, owing to challenges in culturing and detection methods. In clinical settings, the nonculturability of *Lm* poses a significant hurdle to the diagnosis of listeriosis, potentially resulting in underdetection of cases. Interestingly, the detection rate of bacteria in the blood and in the brain is low in cases of neurolisteriosis [8], a phenomenon replicated in a mouse model where neurotropic *Lm* strains reach the brain without being detected in the bloodstream [71].

We provide the first evidence that the divergent fate of *Lm* in microglia and MDM is determined by LLO, which enables *Lm* to escape killing by disrupting the vacuolar membrane and enhances its survival within the harsh conditions of the phagolysosomal apparatus [11, 34]. Infection of microglia with the *hly* deletion mutant phenocopied wild type infection in MDM with the appearance of VBNC forms, while in MDM the rate of bacterial LLO expression was lower and deletion of hly increased the early bacterial killing rate. These results point to differences in bacterial LLO activity between MDM and microglia, the mechanism of which remain to be elucidated.

In conclusion, we unveil the host cell-dependent multifaceted behaviour of *Lm* during neuroinfection. This pathogen thrives within microglia while persisting in MDM in a VBNC state, raising questions about macrophages’ effectiveness in clearing infection. Our data show that *Lm* establishes various host cell-dependent forms of infection reservoirs, likely as a strategy to increase its chances for survival within the host. A better understanding of host cell-dependent infection cycle and intra-macrophage VBNC induction is crucial for the development of tailored therapeutic strategies for neuroinfections aimed at eliminating bacteria and preventing emerging VBNC persisters.

## Supplementary Information

Below is the link to the electronic supplementary material.

Supplementary file1 (PDF 12718 KB)

Supplementary file2 (MOV 191518 KB)

## Data Availability

Data that support the findings of this study have been deposited in the BORIS portal of the University of Bern: https://boris-portal.unibe.ch/handle/20.500.12422/202713.

## References

[1] Abdelhamed H, Lawrence ML, Karsi A (2015) A novel suicide plasmid for efficient gene mutation in *Listeria monocytogenes*. Plasmid 81:1–8. 10.1016/j.plasmid.2015.05.003 26038185 10.1016/j.plasmid.2015.05.003

[2] Andrejewski N, Punnonen E-L, Guhde G, Tanaka Y, Lüllmann-Rauch R, Hartmann D et al (1999) Normal lysosomal morphology and function in LAMP-1-deficient mice. J Biol Chem 274:12692–12701. 10.1074/jbc.274.18.12692 10212251 10.1074/jbc.274.18.12692

[3] Arnaud M, Chastanet A, Débarbouillé M (2004) New vector for efficient allelic replacement in naturally nontransformable, low-GC-content, gram-positive bacteria. Appl Environ Microbiol 70:6887–6891. 10.1128/AEM.70.11.6887-6891.2004 15528558 10.1128/AEM.70.11.6887-6891.2004 PMC525206

[4] Bagatella S, Haghayegh Jahromi N, Monney C, Polidori M, Gall FM, Marchionatti E et al (2022) Bovine neutrophil chemotaxis to *Listeria monocytogenes* in neurolisteriosis depends on microglia-released rather than bacterial factors. J Neuroinflammation 19:304. 10.1186/s12974-022-02653-1 36527076 10.1186/s12974-022-02653-1 PMC9758797

[5] Bagatella S, Monney C, Gross N, Bernier Gosselin V, Schüpbach-Regula G, Hemphill A et al (2025) Intravacuolar persistence in neutrophils facilitates *Listeria monocytogenes* spread to co-cultured cells. MBio 16:e0270024. 10.1128/mbio.02700-24 40067021 10.1128/mbio.02700-24 PMC11980584

[6] Bagatella S, Tavares-Gomes L, Oevermann A (2022) *Listeria monocytogenes* at the interface between ruminants and humans: a comparative pathology and pathogenesis review. Vet Pathol 59:186–210. 10.1177/03009858211052659 34856818 10.1177/03009858211052659

[7] Banović F, Schroten H, Schwerk C (2020) Potential roles and functions of listerial virulence factors during brain entry. Toxins 12:297. 10.3390/toxins12050297 32380697 10.3390/toxins12050297 PMC7291126

[8] Bartt R (2000) *Listeria *and atypical presentations of *Listeria* in the central nervous system. Semin Neurol 20:361–374. 10.1055/s-2000-9398 11051300 10.1055/s-2000-9398

[9] Batista SJ, Still KM, Johanson D, Thompson JA, O’Brien CA, Lukens JR et al (2020) Gasdermin-D-dependent IL-1α release from microglia promotes protective immunity during chronic *Toxoplasma gondii* infection. Nat Commun 11:3687. 10.1038/s41467-020-17491-z 32703941 10.1038/s41467-020-17491-z PMC7378823

[10] Bierne H, Sabet C, Personnic N, Cossart P (2007) Internalins: a complex family of leucine-rich repeat-containing proteins in *Listeria monocytogenes*. Microbes Infect 9:1156–1166. 10.1016/j.micinf.2007.05.003 17764999 10.1016/j.micinf.2007.05.003

[11] Birmingham CL, Canadien V, Kaniuk NA, Steinberg BE, Higgins DE, Brumell JH (2008) Listeriolysin O allows *Listeria monocytogenes* replication in macrophage vacuoles. Nature 451:350–354. 10.1038/nature06479 18202661 10.1038/nature06479

[12] Blériot C, Dupuis T, Jouvion G, Eberl G, Disson O, Lecuit M (2015) Liver-resident macrophage necroptosis orchestrates type 1 microbicidal inflammation and type-2-mediated tissue repair during bacterial infection. Immunity 42:145–158. 10.1016/j.immuni.2014.12.020 25577440 10.1016/j.immuni.2014.12.020

[13] Break TJ, Jun S, Indramohan M, Carr KD, Sieve AN, Dory L (1950) Extracellular superoxide dismutase inhibits innate immune responses and clearance of an intracellular bacterial infection. J Immunol Baltim Md 188:3342–3350. 10.4049/jimmunol.1102341 10.4049/jimmunol.1102341 PMC3311725 22393157

[14] Buchanan RL, Gorris LGM, Hayman MM, Jackson TC, Whiting RC (2017) A review of *Listeria monocytogenes*: an update on outbreaks, virulence, dose-response, ecology, and risk assessments. Food Control 75:1–13. 10.1016/j.foodcont.2016.12.016

[15] Butovsky O, Weiner HL (2018) Microglial signatures and their role in health and disease. Nat Rev Neurosci 19:622–635. 10.1038/s41583-018-0057-5 30206328 10.1038/s41583-018-0057-5 PMC7255106

[16] Chen Z, Zhong D, Li G (2019) The role of microglia in viral encephalitis: a review. J Neuroinflammation 16:76. 10.1186/s12974-019-1443-2 30967139 10.1186/s12974-019-1443-2 PMC6454758

[17] Desjardins M (1995) Biogenesis of phagolysosomes: the ‘kiss and run’ hypothesis. Trends Cell Biol 5:183–186. 10.1016/0962-8924(95)80001-W 14731444 10.1016/s0962-8924(00)88989-8

[18] Di Palma S, Brunetti B, Doherr MG, Forster U, Hilbe M, Zurbriggen A et al (2012) Comparative spatiotemporal analysis of the intrathecal immune response in natural listeric rhombencephalitis of cattle and small ruminants. Comp Immunol Microbiol Infect Dis 35:429–441. 10.1016/j.cimid.2012.03.009 22537479 10.1016/j.cimid.2012.03.009

[19] DiSabato D, Quan N, Godbout JP (2016) Neuroinflammation: the devil is in the details. J Neurochem 139:136–153. 10.1111/jnc.13607 26990767 10.1111/jnc.13607 PMC5025335

[20] Drevets DA, Canono BP, Campbell PA (2015) Measurement of bacterial ingestion and killing by macrophages. Curr Protoc Immunol. 10.1002/0471142735.im1406s109 18432724 10.1002/0471142735.im1406s12

[21] Drummond RA, Swamydas M, Oikonomou V, Zhai B, Dambuza IM, Schaefer BC et al (2019) CARD9+ microglia promote antifungal immunity via IL-1β- and CXCL1-mediated neutrophil recruitment. Nat Immunol 20:559–570. 10.1038/s41590-019-0377-2 30996332 10.1038/s41590-019-0377-2 PMC6494474

[22] Esche C, Stellato C, Beck LA (2005) Chemokines: key players in innate and adaptive immunity. J Invest Dermatol 125:615–628. 10.1111/j.0022-202X.2005.23841.x 16185259 10.1111/j.0022-202X.2005.23841.x

[23] Eskelinen E-L (2006) Roles of LAMP-1 and LAMP-2 in lysosome biogenesis and autophagy. Mol Aspects Med 27:495–502. 10.1016/j.mam.2006.08.005 16973206 10.1016/j.mam.2006.08.005

[24] Fleming SD, Campbell PA (1997) Some macrophages kill *Listeria monocytogenes* while others do not. Immunol Rev 158:69–77. 10.1111/j.1600-065X.1997.tb00993.x 9314075 10.1111/j.1600-065x.1997.tb00993.x

[25] Frande-Cabanes E, Fernandez-Prieto L, Calderon-Gonzalez R, Rodríguez-Del Río E, Yañez-Diaz S, López-Fanarraga M et al (2014) Dissociation of innate immune responses in microglia infected with *Listeria monocytogenes*. Glia 62:233–246. 10.1002/glia.22602 24311463 10.1002/glia.22602 PMC4068285

[26] Gedde MM, Higgins DE, Tilney LG, Portnoy DA (2000) Role of listeriolysin O in cell-to-cell spread of *Listeria monocytogenes*. Infect Immun 68:999–1003. 10.1128/IAI.68.2.999-1003.2000 10639481 10.1128/iai.68.2.999-1003.2000 PMC97240

[27] Glomski IJ, Decatur AL, Portnoy DA (2003) *Listeria monocytogenes* mutants that fail to compartmentalize listerolysin O activity are cytotoxic, avirulent, and unable to evade host extracellular defenses. Infect Immun 71:6754–6765. 10.1128/IAI.71.12.6754-6765.2003 14638761 10.1128/IAI.71.12.6754-6765.2003 PMC308949

[28] Gluschko A, Herb M, Wiegmann K, Krut O, Neiss WF, Utermöhlen O et al (2018) The β2 integrin Mac-1 induces protective LC3-associated phagocytosis of *Listeria monocytogenes*. Cell Host Microbe 23:324-337.e5. 10.1016/j.chom.2018.01.018 29544096 10.1016/j.chom.2018.01.018

[29] Gözel B, Monney C, Aguilar-Bultet L, Rupp S, Frey J, Oevermann A (2019) Hyperinvasiveness of *Listeria monocytogenes* sequence type 1 is independent of lineage I-specific genes encoding internalin-like proteins. MicrobiologyOpen. 10.1002/mbo3.790 30656829 10.1002/mbo3.790 PMC6612545

[30] Gray ML, Stafseth HJ (1948) A new technique for isolating *Listerellae* from the bovine brain. J Bacteriol 55:471–476. 10.1128/JB.55.4.471-476.1948 18909073 10.1128/JB.55.4.471-476.1948

[31] Guldimann C, Bärtschi M, Frey J, Zurbriggen A, Seuberlich T, Oevermann A (2015) Increased spread and replication efficiency of *Listeria monocytogenes* in organotypic brain-slices is related to multilocus variable number of tandem repeat analysis (MLVA) complex. BMC Microbiol 15:134. 10.1186/s12866-015-0454-0 26138984 10.1186/s12866-015-0454-0 PMC4490720

[32] Guldimann C, Lejeune B, Hofer S, Leib SL, Frey J, Zurbriggen A et al (2012) Ruminant organotypic brain-slice cultures as a model for the investigation of CNS listeriosis. Int J Exp Pathol 93:259–268. 10.1111/j.1365-2613.2012.00821.x 22804762 10.1111/j.1365-2613.2012.00821.x PMC3444982

[33] Hafner L, Pichon M, Burucoa C, Nusser SHA, Moura A, Garcia-Garcera M et al (2021) *Listeria monocytogenes* faecal carriage is common and depends on the gut microbiota. Nat Commun 12:6826. 10.1038/s41467-021-27069-y 34819495 10.1038/s41467-021-27069-y PMC8613254

[34] Hamon MA, Ribet D, Stavru F, Cossart P (2012) Listeriolysin O: the Swiss army knife of Listeria. Trends Microbiol 20:360–368. 10.1016/j.tim.2012.04.006 22652164 10.1016/j.tim.2012.04.006

[35] Henke D, Rupp S, Gaschen V, Stoffel MH, Frey J, Vandevelde M et al (2015) *Listeria monocytogenes* spreads within the brain by actin-based intra-axonal migration. Infect Immun 83:2409–2419. 10.1128/IAI.00316-15 25824833 10.1128/IAI.00316-15 PMC4432752

[36] Herb M, Gluschko A, Schramm M (2020) LC3-associated phagocytosis—the highway to hell for phagocytosed microbes. Semin Cell Dev Biol 101:68–76. 10.1016/j.semcdb.2019.04.016 31029766 10.1016/j.semcdb.2019.04.016

[37] Huang J, Brumell JH (2014) Bacteria–autophagy interplay: a battle for survival. Nat Rev Microbiol 12:101–114. 10.1038/nrmicro3160 24384599 10.1038/nrmicro3160 PMC7097477

[38] Johnson MB, Criss AK (2013) Fluorescence microscopy methods for determining the viability of bacteria in association with mammalian cells. J Vis Exp. 10.3791/50729 24056524 10.3791/50729 PMC3814296

[39] Jungi TW, Pfister H, Sager H, Fatzer R, Vandevelde M, Zurbriggen A (1997) Comparison of inducible nitric oxide synthase expression in the brains of *Listeria monocytogenes*-infected cattle, sheep, and goats and in macrophages stimulated in vitro. Infect Immun 65:5279–5288 9393827 10.1128/iai.65.12.5279-5288.1997 PMC175760

[40] Kazmi F, Hensley T, Pope C, Funk RS, Loewen GJ, Buckley DB et al (2013) Lysosomal sequestration (trapping) of lipophilic amine (cationic amphiphilic) drugs in immortalized human hepatocytes (Fa2N-4 Cells). Drug Metab Dispos 41:897–905. 10.1124/dmd.112.050054 23378628 10.1124/dmd.112.050054 PMC3608459

[41] Kortebi M, Milohanic E, Mitchell G, Péchoux C, Prevost M-C, Cossart P et al (2017) *Listeria monocytogenes* switches from dissemination to persistence by adopting a vacuolar lifestyle in epithelial cells. PLoS Pathog 13:e1006734. 10.1371/journal.ppat.1006734 29190284 10.1371/journal.ppat.1006734 PMC5708623

[42] Lamason RL, Welch MD (2017) Actin-based motility and cell-to-cell spread of bacterial pathogens. Curr Opin Microbiol 35:48–57. 10.1016/j.mib.2016.11.007 27997855 10.1016/j.mib.2016.11.007 PMC5474209

[43] Lawson LJ, Perry VH, Dri P, Gordon S (1990) Heterogeneity in the distribution and morphology of microglia in the normal adult mouse brain. Neuroscience 39:151–170. 10.1016/0306-4522(90)90229-W 2089275 10.1016/0306-4522(90)90229-w

[44] Lecuit M (2005) Understanding how *Listeria monocytogenes* targets and crosses host barriers. Clin Microbiol Infect 11:430–436. 10.1111/j.1469-0691.2005.01146.x 15882192 10.1111/j.1469-0691.2005.01146.x

[45] Lee H-J, Woo Y, Hahn T-W, Jung YM, Jung Y-J (2020) Formation and maturation of the phagosome: a key mechanism in innate immunity against intracellular bacterial infection. Microorganisms. 10.3390/microorganisms8091298 32854338 10.3390/microorganisms8091298 PMC7564318

[46] Levin R, Grinstein S, Canton J (2016) The life cycle of phagosomes: formation, maturation, and resolution. Immunol Rev 273:156–179. 10.1111/imr.12439 27558334 10.1111/imr.12439

[47] Li Q, Barres BA (2018) Microglia and macrophages in brain homeostasis and disease. Nat Rev Immunol 18:225–242. 10.1038/nri.2017.125 29151590 10.1038/nri.2017.125

[48] Lotoux A, Milohanic E, Bierne H (2022) The viable but non-culturable state of *Listeria monocytogenes* in the one-health continuum. Front Cell Infect Microbiol. 10.3389/fcimb.2022.849915 35372114 10.3389/fcimb.2022.849915 PMC8974916

[49] Maury MM, Bracq-Dieye H, Huang L, Vales G, Lavina M, Thouvenot P et al (2019) Hypervirulent *Listeria monocytogenes* clones’ adaption to mammalian gut accounts for their association with dairy products. Nat Commun 10:2488. 10.1038/s41467-019-10380-0 31171794 10.1038/s41467-019-10380-0 PMC6554400

[50] Meunier E, Broz P (2015) Quantification of cytosolic vs. vacuolar *Salmonella* in primary macrophages by differential permeabilization. J Vis Exp. 10.3791/52960 26274778 10.3791/52960 PMC4545148

[51] Mohamed SH, Fu MS, Hain S, Alselami A, Vanhoffelen E, Li Y et al (2023) Microglia are not protective against cryptococcal meningitis. Nat Commun 14:7202. 10.1038/s41467-023-43061-0 37938547 10.1038/s41467-023-43061-0 PMC10632471

[52] Moran J, Feltham L, Bagnall J, Goldrick M, Lord E, Nettleton C et al (2023) Live-cell imaging reveals single-cell and population-level infection strategies of *Listeria monocytogenes* in macrophages. Front Immunol 14:1235675. 10.3389/fimmu.2023.1235675 37675103 10.3389/fimmu.2023.1235675 PMC10478088

[53] Müller J, Aguado A, Laleu B, Balmer V, Ritler D, Hemphill A (2017) In vitro screening of the open source Pathogen Box identifies novel compounds with profound activities against *Neospora caninum*. Int J Parasitol 47:801–809. 10.1016/j.ijpara.2017.06.002 28751177 10.1016/j.ijpara.2017.06.002

[54] Myers JT, Tsang AW (1950) Localized reactive oxygen and nitrogen intermediates inhibit escape of *Listeria monocytogenes* from vacuoles in activated macrophages. J Immunol Baltim Md 171:5447–5453. 10.4049/jimmunol.171.10.5447 10.4049/jimmunol.171.10.5447 PMC2972186 14607950

[55] Oevermann A, Di Palma S, Doherr MG, Abril C, Zurbriggen A, Vandevelde M (2010) Neuropathogenesis of naturally occurring encephalitis caused by *Listeria monocytogenes* in ruminants. Brain Pathol Zurich Switz 20:378–390. 10.1111/j.1750-3639.2009.00292.x 10.1111/j.1750-3639.2009.00292.x PMC8094665 19476464

[56] Oevermann A, Zurbriggen A, Vandevelde M (2010) Rhombencephalitis caused by *Listeria monocytogenes* in humans and ruminants: a zoonosis on the rise? Interdiscip Perspect Infect Dis. 10.1155/2010/632513 20204066 10.1155/2010/632513 PMC2829626

[57] Oliver JD (2010) Recent findings on the viable but nonculturable state in pathogenic bacteria. FEMS Microbiol Rev 34:415–425. 10.1111/j.1574-6976.2009.00200.x 20059548 10.1111/j.1574-6976.2009.00200.x

[58] Paiva CN, Bozza MT (2013) Are reactive oxygen species always detrimental to pathogens? Antioxid Redox Signal 20:1000–1037. 10.1089/ars.2013.5447 23992156 10.1089/ars.2013.5447 PMC3924804

[59] Peron-Cane C, Fernandez J-C, Leblanc J, Wingertsmann L, Gautier A, Desprat N et al (2020) Fluorescent secreted bacterial effectors reveal active intravacuolar proliferation of *Listeria monocytogenes* in epithelial cells. PLoS Pathog. 10.1371/journal.ppat.1009001 33045003 10.1371/journal.ppat.1009001 PMC7580998

[60] Personnic N, Doublet P, Jarraud S (2023) Intracellular persister: A stealth agent recalcitrant to antibiotics. Front Cell Infect Microbiol. 10.3389/fcimb.2023.1141868 37065203 10.3389/fcimb.2023.1141868 PMC10102521

[61] Peyrusson F, Nguyen TK, Najdovski T, Van Bambeke F (2022) Host cell oxidative stress induces dormant *Staphylococcus aureus* persisters. Microbiol Spectr 10:e02313–e2321. 10.1128/spectrum.02313-21 35196815 10.1128/spectrum.02313-21 PMC8865412

[62] Pizarro-Cerdá J, Cossart P (2018) *Listeria monocytogenes*: cell biology of invasion and intracellular growth. Microbiol Spectr. 10.1128/microbiolspec.GPP3-0013-2018 30523778 10.1128/microbiolspec.gpp3-0013-2018 PMC11633638

[63] Portnoy DA, Chen C, Mitchell G (2016) Strategies used by bacteria to grow in macrophages. Microbiol Spectr. 10.1128/microbiolspec.MCHD-0012-2015 27337444 10.1128/microbiolspec.MCHD-0012-2015 PMC4922531

[64] Portnoy DA, Schreiber RD, Connelly P, Tilney LG (1989) Gamma interferon limits access of *Listeria monocytogenes* to the macrophage cytoplasm. J Exp Med 170:2141–2146. 10.1084/jem.170.6.2141 2511268 10.1084/jem.170.6.2141 PMC2189551

[65] Radoshevich L, Cossart P (2018) *Listeria monocytogenes*: towards a complete picture of its physiology and pathogenesis. Nat Rev Microbiol 16:32–46. 10.1038/nrmicro.2017.126 29176582 10.1038/nrmicro.2017.126

[66] Ricci A, Allende A, Bolton D, Chemaly M, Davies R, Fernández Escámez PS et al (2018) *Listeria monocytogenes* contamination of ready-to-eat foods and the risk for human health in the EU. EFSA J 16:e05134. 10.2903/j.efsa.2018.5134 32760461 10.2903/j.efsa.2018.5134 PMC7391409

[67] Rupp S, Bärtschi M, Frey J, Oevermann A (2017) Hyperinvasiveness and increased intercellular spread of *Listeria monocytogenes *sequence type 1 are independent of listeriolysin S, internalin F and internalin J1. J Med Microbiol 66:1053–1062. 10.1099/jmm.0.000529 28708050 10.1099/jmm.0.000529

[68] Schafer DP, Stevens B (2015) Microglia function in central nervous system development and plasticity. Cold Spring Harb Perspect Biol 7:a020545. 10.1101/cshperspect.a020545 26187728 10.1101/cshperspect.a020545 PMC4588063

[69] Schindelin J, Arganda-Carreras I, Frise E, Kaynig V, Longair M, Pietzsch T et al (2012) Fiji: an open-source platform for biological-image analysis. Nat Methods 9:676–682. 10.1038/nmeth.2019 22743772 10.1038/nmeth.2019 PMC3855844

[70] Schlech WF III, Acheson D (2000) Foodborne listeriosis. Clin Infect Dis 31:770–775. 10.1086/314008 11017828 10.1086/314008

[71] Senay TE, Ferrell JL, Garrett FG, Albrecht TM, Cho J, Alexander KL et al (2020) Neurotropic lineage III strains of *Listeria monocytogenes* disseminate to the brain without reaching high titer in the blood. mSphere 5:e00871–e920. 10.1128/mSphere.00871-20 32938704 10.1128/mSphere.00871-20 PMC7494839

[72] Sevenich L (2018) Brain-resident microglia and blood-borne macrophages orchestrate central nervous system inflammation in neurodegenerative disorders and brain cancer. Front Immunol. 10.3389/fimmu.2018.00697 29681904 10.3389/fimmu.2018.00697 PMC5897444

[73] Shaughnessy LM (2007) The role of the activated macrophage in clearing *Listeria monocytogenes* infection. Front Biosci 12:2683. 10.2741/2364 17127272 10.2741/2364 PMC2851543

[74] Shaughnessy LM, Hoppe AD, Christensen KA, Swanson JA (2006) Membrane perforations inhibit lysosome fusion by altering pH and calcium in *Listeria monocytogenes* vacuoles. Cell Microbiol 8:781–792. 10.1111/j.1462-5822.2005.00665.x 16611227 10.1111/j.1462-5822.2005.00665.x PMC1435990

[75] Tan Y-L, Yuan Y, Tian L (2020) Microglial regional heterogeneity and its role in the brain. Mol Psychiatry 25:351–367. 10.1038/s41380-019-0609-8 31772305 10.1038/s41380-019-0609-8 PMC6974435

[76] Tavares-Gomes L, Monney C, Neuhaus G, Francisco D, Solis D, Summerfield A et al (2021) Transcriptome of microglia reveals a species-specific expression profile in bovines with conserved and new signature genes. Glia 69:1932–1949. 10.1002/glia.24002 33811399 10.1002/glia.24002

[77] Tran TT, Mathmann CD, Gatica-Andrades M, Rollo RF, Oelker M, Ljungberg JK et al (2022) Inhibition of the master regulator of *Listeria monocytogenes* virulence enables bacterial clearance from spacious replication vacuoles in infected macrophages. PLoS Pathog 18:e1010166. 10.1371/journal.ppat.1010166 35007292 10.1371/journal.ppat.1010166 PMC8746789

[78] Tucker JS, Cho J, Albrecht TM, Ferrell JL, D’Orazio SEF (2023) Egress of *Listeria monocytogenes* from mesenteric lymph nodes depends on intracellular replication and cell-to-cell spread. Infect Immun 91:e0006423. 10.1128/iai.00064-23 36916918 10.1128/iai.00064-23 PMC10112146

[79] Uribe-Querol E, Rosales C (2017) Control of phagocytosis by microbial pathogens. Front Immunol. 10.3389/fimmu.2017.01368 29114249 10.3389/fimmu.2017.01368 PMC5660709

[80] Wang Z, Sun D, Chen G, Li G, Dou S, Wang R et al (2017) Tim-3 inhibits macrophage control of *Listeria monocytogenes* by inhibiting Nrf2. Sci Rep 7:42095. 10.1038/srep42095 28205579 10.1038/srep42095 PMC5311873

[81] Welch MD, Rosenblatt J, Skoble J, Portnoy DA, Mitchison TJ (1998) Interaction of human Arp2/3 complex and the *Listeria monocytogenes* acta protein in actin filament nucleation. Science 281:105–108. 10.1126/science.281.5373.105 9651243 10.1126/science.281.5373.105

[82] Whiteley AT, Ruhland BR, Edrozo MB, Reniere ML (2017) A redox-responsive transcription factor is critical for pathogenesis and aerobic growth of *Listeria monocytogenes*. Infect Immun. 10.1128/IAI.00978-16 28193635 10.1128/IAI.00978-16 PMC5400837

[83] Yamasaki R, Lu H, Butovsky O, Ohno N, Rietsch AM, Cialic R et al (2014) Differential roles of microglia and monocytes in the inflamed central nervous system. J Exp Med 211:1533–1549. 10.1084/jem.20132477 25002752 10.1084/jem.20132477 PMC4113947

