# Supplementary material for: Divergent host–pathogen interactions in neurolisteriosis: cytosolic replication vs. phagosomal dormancy of Listeria monocytogenes in CNS macrophages
Source: Acta Neuropathol. 2025 Jun 16;149(1):63. doi: 10.1007/s00401-025-02900-8 (PMC12170700; doi:10.1007/s00401-025-02900-8)
Supplement: Supplementary file 1 — Supplementary file1 (PDF 12718 KB) [file 401_2025_2900_MOESM1_ESM.pdf]

Supplementary Information (SI)

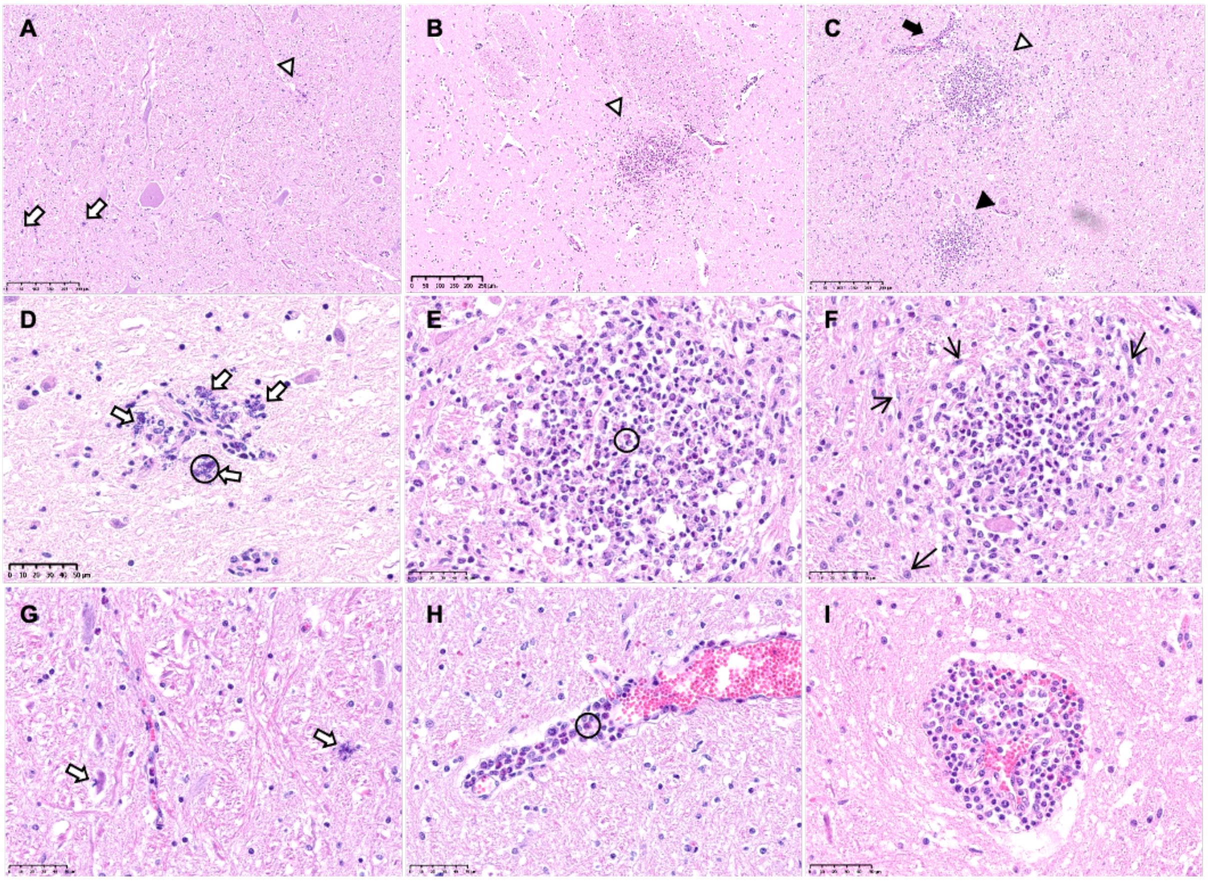

SI, Fig S1. H&E images from bovine neurolisteriosis cases. A: Midbrain area of the case shown in Figure 1 A-C, showing an early microabscess (arrowhead). The microabscess is not surrounded by perivascular inflammatory infiltrates. Bacterial colonies are indicated by white arrows. B: Brainstem region containing the type II microabscess (arrowhead) shown in Figure 1A-C. C: Brainstem region containing an acute/subacute (type II, arrowhead) and a chronic (type III, black arrowhead) microabscess located near each other, along with a perivascular cuff (black arrow). D: Magnified view of the type I microabscess from (A), showing a small lesion (circular area= 0.0113 mm^2) containing few phagocytes, including ameboid microglia-like cells and scattered neutrophils (circled). *Lm* colonies (white arrows) are associated with mononuclear phagocytes, compatible with microglia-like cells. The surrounding neuropil

appears oedematous. E: Magnified view of the type II microabscesses from (B), with a circular area of 0.075 mm^2. The lesion contains abundant polymorphonucleated cells (neutrophils, circled) in the center. F: Magnified view of the type III microabscesses from (C) (circular area=0.0699 mm^2), showing a predominance of MDM-like cells, with fewer intralesional neutrophils and reactive astrocytes (astrogliosis, black arrows). G: Magnified view of (A), showing bacterial colonies either associated with neurons or freely distributed within the neuropil (white arrows). Nearby perivascular spaces lack perivascular cuffs. H: Perivascular cuff adjacent to a type II microabscess, composed of neutrophils (circled), MDM, and lymphocytes. I: Perivascular cuff near a type III microabscess, consisting primarily of MDM and lymphocytes, with sparse neutrophils.

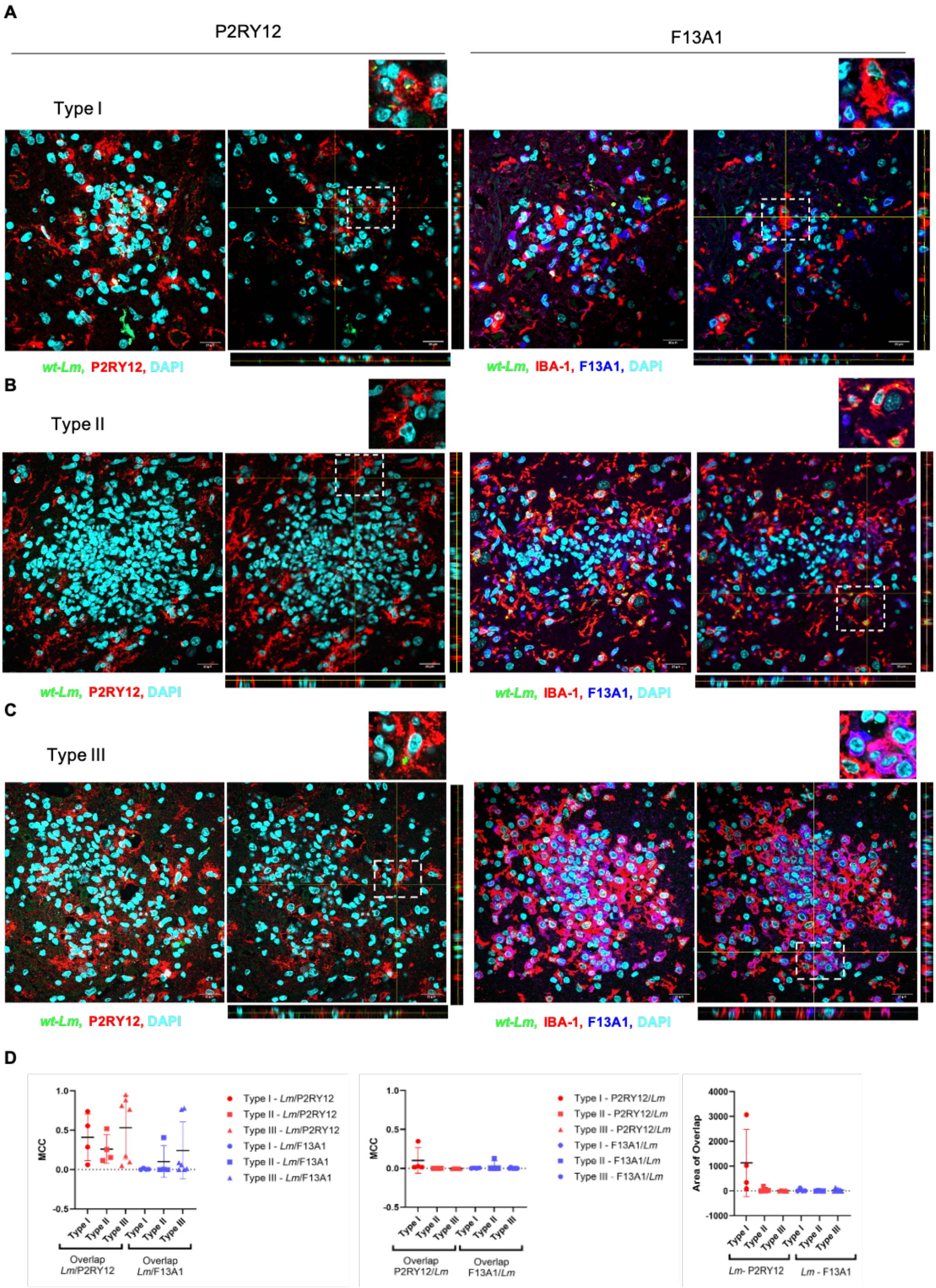

SI, Fig S2. Association between *Lm* and microglia and MDM across different types of microabscesses. Fig. A-C: Confocal images of representative type I-III microabscesses from

serial sections, stained with either P2RY12 and *Lm* (left panels) or F13A1, IBA-1, and *Lm* (right panels). In each panel, the left image shows a maximum intensity projection from the Z-stack, while the right image displays orthogonal views of a single optical slice. Insets at the top right of each panel provide magnified views of the areas outlined by white boxes.

A: Type I microabscess showing single or multiple intracytoplasmic *Lm* localized within P2RY12+ (left panel) or IBA-1+F13A1- (right panel) microglia, or outside microglia but not within F13A1+ MDMs. Fig. B: Type II microabscess, in which *Lm* are predominantly found as single or multiple bacteria within the cytoplasm of P2RY12+ and IBA1+F13A1- microglia at the lesion periphery. Fig. C: Confocal images of a type III microabscess with insets, showing *Lm* within a microglial cell (left) or associated with an IBA1+F13A1+ MDM and an unlabelled cell (right). D: Manders' colocalization coefficients (MCCs) quantifying the overlap between *Lm* fluorescence and either P2RY12 or F13A1 (left panel), or between either P2RY12 or F13A1 and *Lm* (middle panel). E: Area of overlap (µm^2) between *Lm* and either P2RY12 or F13A1. Datapoints in D and E represent MCCs, and areas of overlap obtained from each microabscess in consecutive sections co-stained with either anti-P2RY12 and anti-*Lm* antibodies (red) or with anti-F13A1 and anti-*Lm* antibodies (blue), grouped by microabscess type. Analyses were performed only on microabscesses containing detectable bacteria (4/5 type I, 4/12 type II, and 7/35 type III).

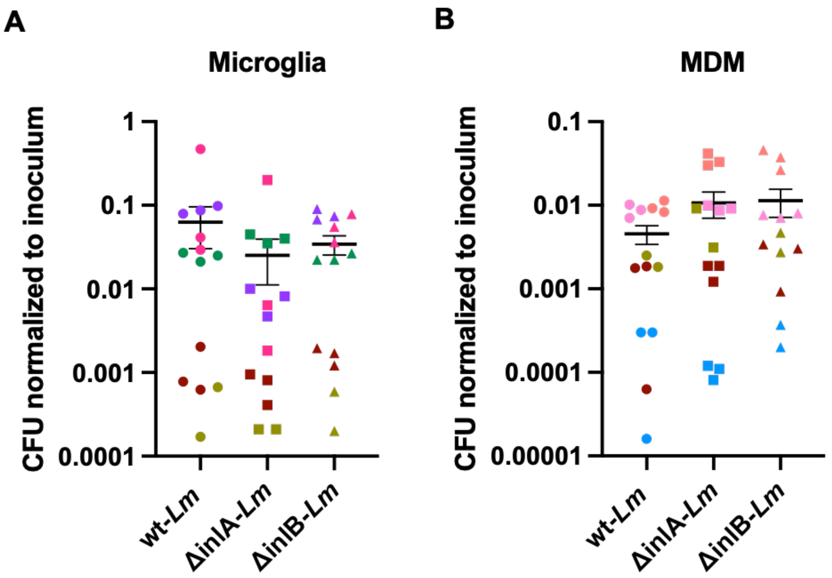

SI, Fig S3. Deletion of *inlA* and *inlB* does not affect *Lm* invasion rate and growth dynamics. Quantification of CFU in the gentamicin protection assay using **a,** primary bovine microglia and **b,** monocyte-derived macrophages (MDM) infected with JF5203-wt *Lm* and with bacterial *inlA* and *inlB* deletion mutants (JF5203-Δ*inlA* and JF5203-Δ*inlB*, respectively) at early time points of infection (45 minutes). Statistical analysis using the Mann-Whitney U test showed no evidence of a difference between wt and the deletion mutants at 45 minutes in microglia or MDM (n = 5 per group, 4 in technical triplicates, 1 in duplicates).

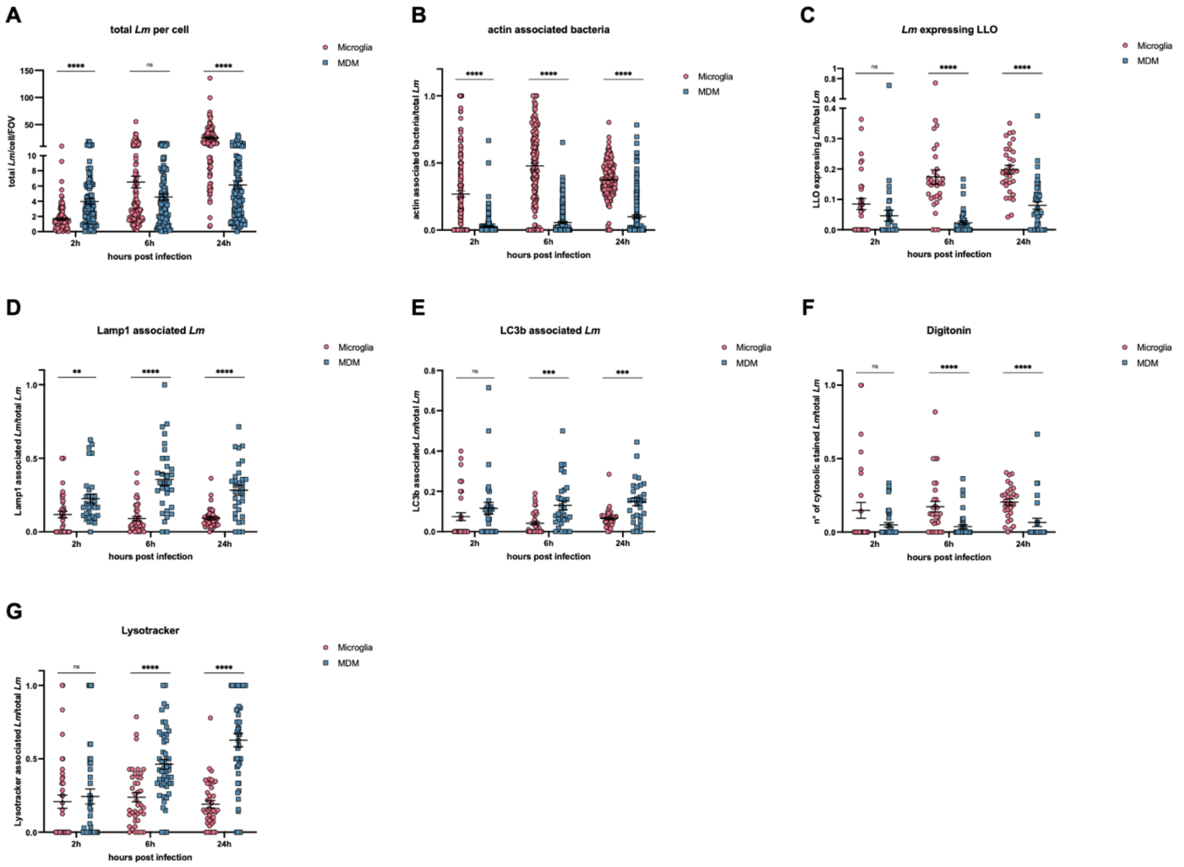

SI, Fig S4. Quantification of Immunofluorescence (IF) pictures per field of view. Gentamicin protection assays analysed by IF. Manual quantification (using the CellCounter plugin in FIJI) per field of view (FOV) of: **a,** total *Listeria monocytogenes* (*Lm*) per cell; **b,** actin associated bacteria per total *Lm*; **c,** LLO expressing bacteria per total *Lm*; **d,** Lamp1 associated bacteria per total *Lm*; **e,** LC3b associated bacteria per total *Lm*; **f,** number of cytosolic bacteria per total *Lm*; **g,** Lysotracker associated bacteria per total *Lm* at 2h, 6h and 24h post-infection. Each data point represents one picture. The bacterial quantifications are based on at least 3 biological replicates per cell type and condition, with 10 images analysed per time point and condition. Error bars indicate mean with SEM, ns: not significant, ** $p < 0.01$, *** $p < 0.001$ and **** $p < 0.0001$ (Mann-Whitney U test).

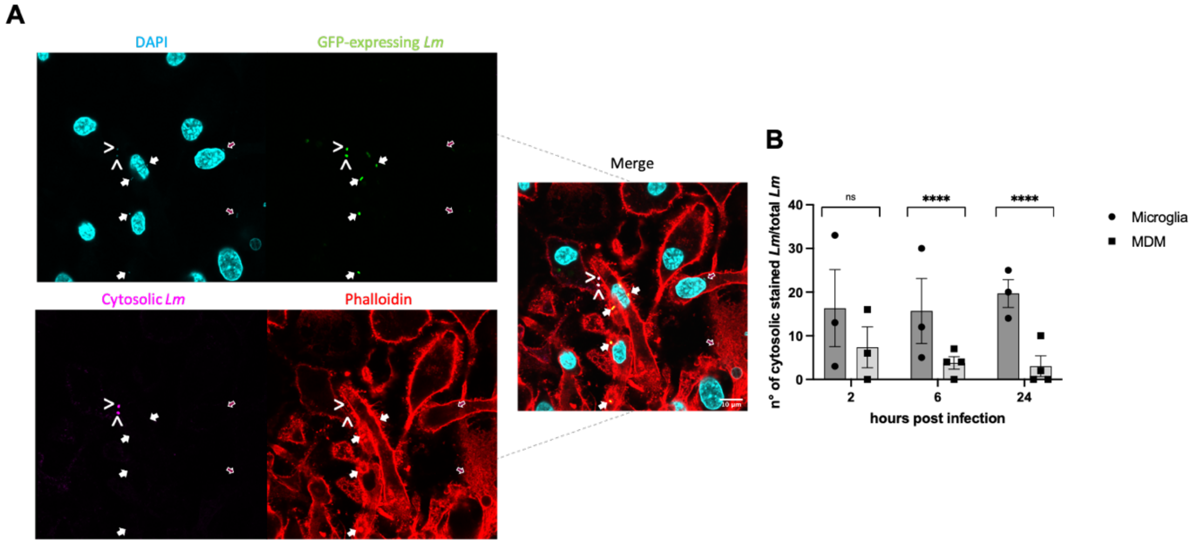

SI, Fig S5. Percentage of cytosolic bacteria is higher in microglia than in MDM.

a, Representative images of microglia infected with JF5203-wt *Lm* at 6h post infection (pi) using a phagosome protection assay. Due to the selective permeabilization of the cytoplasmic membrane, cytosolic *Lm* exhibit dual fluorescence (green for GFP and pink for the *Lm* antibody; white arrowheads), while intravacuolar *Lm* are only fluorescent in green. Notably, the *Lm* antibody signal (pink) is absent in cytosolic bacteria surrounded by polymerized cellular actin (white solid arrows), despite their cytosolic localization as indicated by actin polymerization. Purple arrows indicate intravacuolar bacteria with faded GFP signal that are not detected by the *Lm* antibody. **b,** Quantification of intravacuolar *versus* intracytosolic bacteria, revealing a significantly higher proportion of cytosolic *Lm* in microglia compared to MDM, despite the underestimation of cytosolic bacteria by the *Lm* antibody stain. Analysis was performed using a minimum of 10 images per time point in each of three independent experiments. Error bars indicate SEM, ns: not significant and **** $p < 0.0001$ (Mann-Whitney U test performed in absolute numbers (SI Appendix, Fig. S4)).

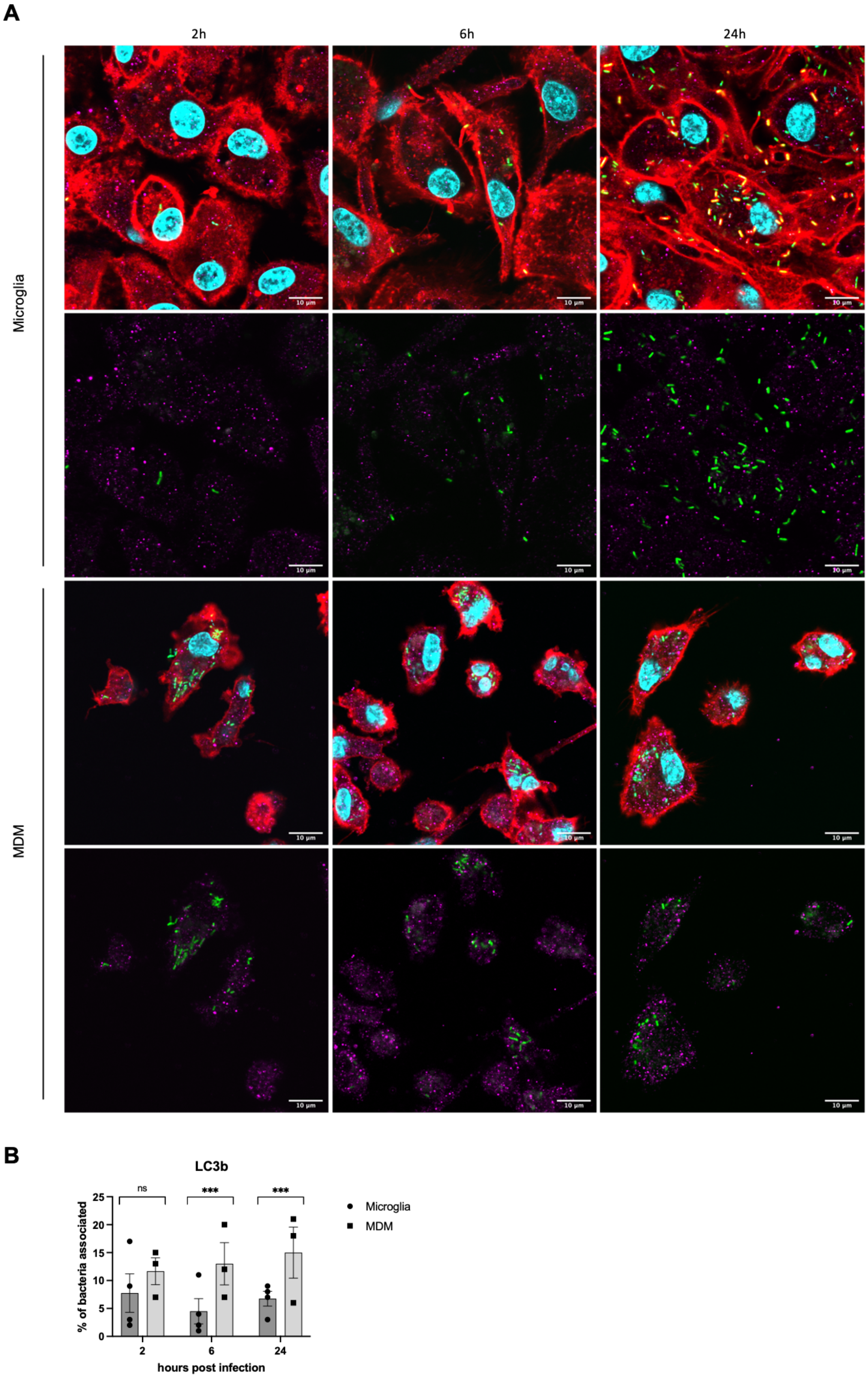

A
2h
6h
24h
Microglia
MDM
10 µm
B
LC3b
ns
***
***
% of bacteria associated
25
20
15
10
5
0
2
6
24
hours post infection
Microglia
MDM

SI, Fig S6. **Microglia and MDM exhibits a minor proportion of bacteria entering the LAP pathway, with comparatively higher levels of bacterial association with LC3b in MDM.** **a,** Representative images illustrating the bacterial association with LC3b (pink) in microglia and MDM at 2, 6 and 24h post infection (pi). The upper panel shows merged images with cellular nuclei stained in cyan (DAPI), GFP expressing JF5203-wt *Lm* in green, cellular actin in red (phalloidin), and LC3b in pink. The lower panel shows LC3b in pink and GFP expressing JF5203-wt *Lm* in green. **b,** Percentage of bacteria associated with LC3b in microglia and MDM at 2, 6 and 24h pi is lower than bacteria associated with Lamp1 (Fig. 3). The fraction of LC3b-associated bacteria is slightly higher in MDM than in microglia. Each data point represents an individual experiment, using primary cells obtained from individual animals for each cell type. A minimum of 10 images were acquired per time point, per experiment resulting containing a total of 6'517 bacteria. Error bars indicate SEM, ns: not significant and *** $p < 0.001$ (Mann-Whitney U test performed in absolute numbers (SI Appendix, Fig. S4)).

A

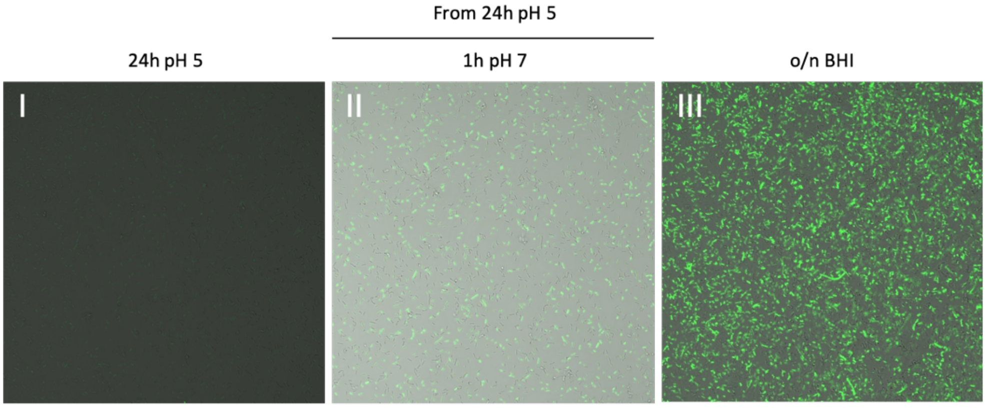

B

	GFP level expression after different pH exposition								
	**pH 2**	**pH 3**	**pH 4**	**pH 5**	pH 6	pH 7	pH 8	pH 9	pH 10
30 min	0	0	1	2	3	4	4	3	3
2h		0	1	3	3	4	3	3	3
6h		0	0	3	4	4	4	4	4
24h	0		0	0	2	4	4	4	4
o/n BHI	0<br>-	0<br>+	1<br>++	4<br>++					
	Change to pH 7								
26h	0	0	0	3					
27h	0	0	0	3					
30h	0	0	0	3					
48h	0	0	0	3					

***0**: no GFP; **1**: very low; **2**: low;*
***3**: moderate; **4**: normal*
- no growth; + very little grow;
++ normal bacterial growth

SI, Fig S7 Exposure to pH5 for 24 hours nearly abolishes the GFP signal but not bacterial growth of JF5203-wt *Lm*. a, immunofluorescence images (IF) of GFP-expressing *Lm* showing (I) quenching of GFP-signal after 24h of exposure to pH5, (II) recovery of GFP signal after 1h transfer to pH7, and (III) rest oration of normal GFP signal after overnight (ON) culture in BHI. **b,** Semiquantitative expression levels of GFP at different culture time points (30 min, 2h, 6h and 24h) across pH values ranging from 2 to 10. Expression levels were categorized as follows based on IF pictures: 0= no signal, 1= very low signal, 2= low signal, 3= moderate signal and 4= normal levels. To assess bacteria recovery of GFP signal, bacteria exposed to pH's ranging 2 to 5 were transferred to pH7. Only bacteria exposed to pH5 recovered GFP signal. Viability and growth of bacteria exposed to pH2-5 were assessed by ON culture in BHI medium at pH7, demonstrating that bacterial viability was only affected at pH3 or lower. -: no growth, +: little growth, ++: normal bacterial growth.

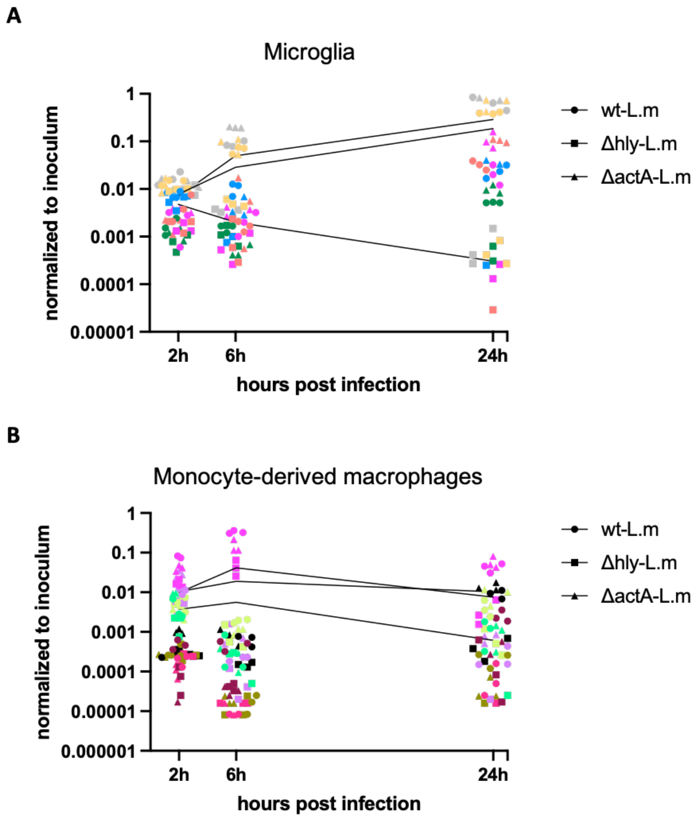

SI, Fig S8. Effect of *hly* and *actA* deletion on infection dynamics in microglia and MDM across individual animals.

Data from Figure 5, broken down by individual animals, showing CFU quantification of intracellular bacteria in microglia (A) and MDMs (B) at 2h, 6h and 24h post infection (pi). Cells from individual calves are represented in different colors, while symbol shapes denote the bacterial strain. Data refers to 6 (A) and 8 (B) individual animals performed in 3 technical replicates.

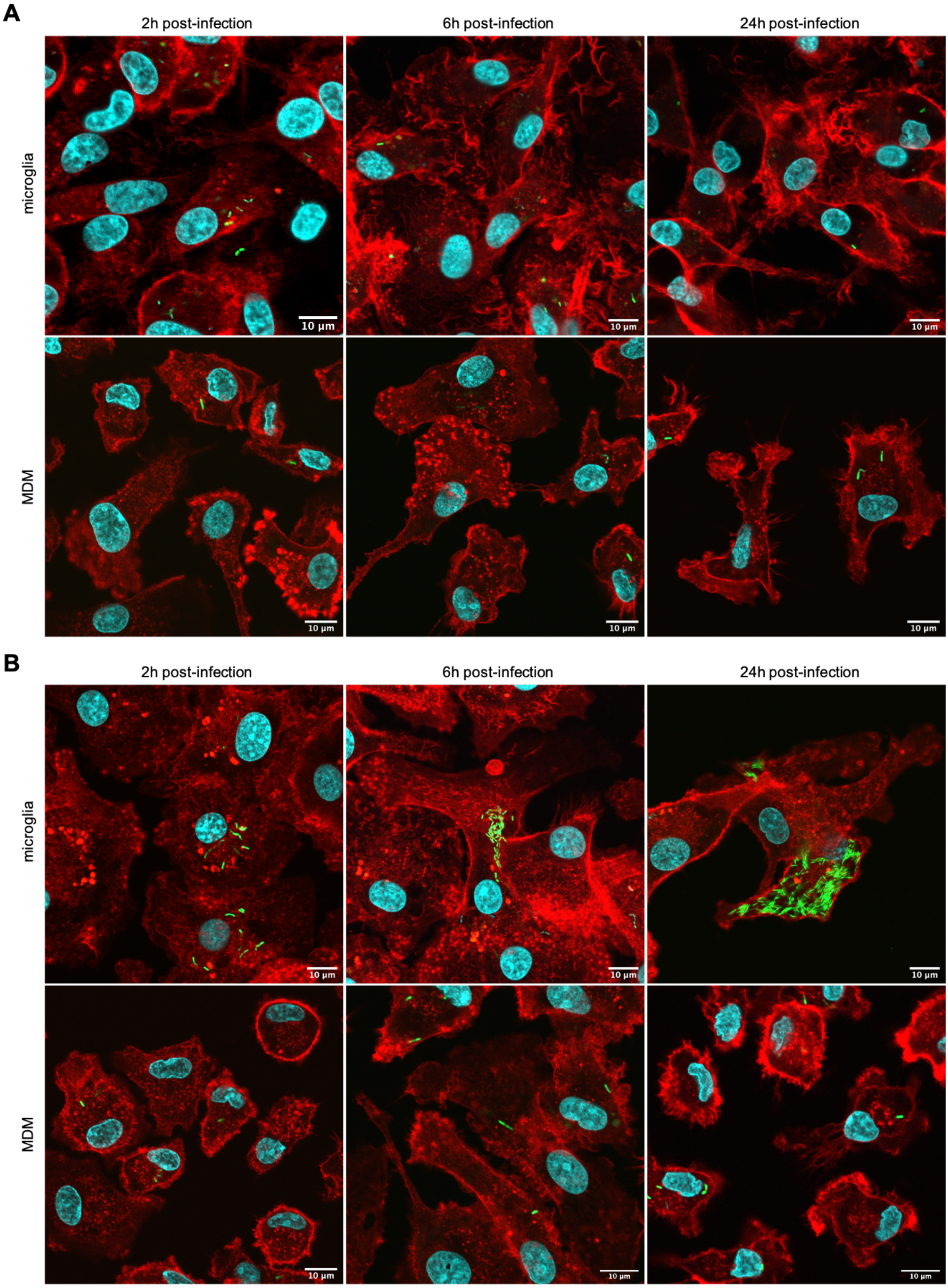

SI, Fig S9. Distinct infection phenotype of *hly* and *actA* deletion mutants in microglia and MDM. Representative images of infection phenotype of *Lm* mutants in microglia and MDM: **a,** JF5203-Δ*hly* and **b,** JF5203-Δ*actA* at 2h, 6h and 24 hours post infection.

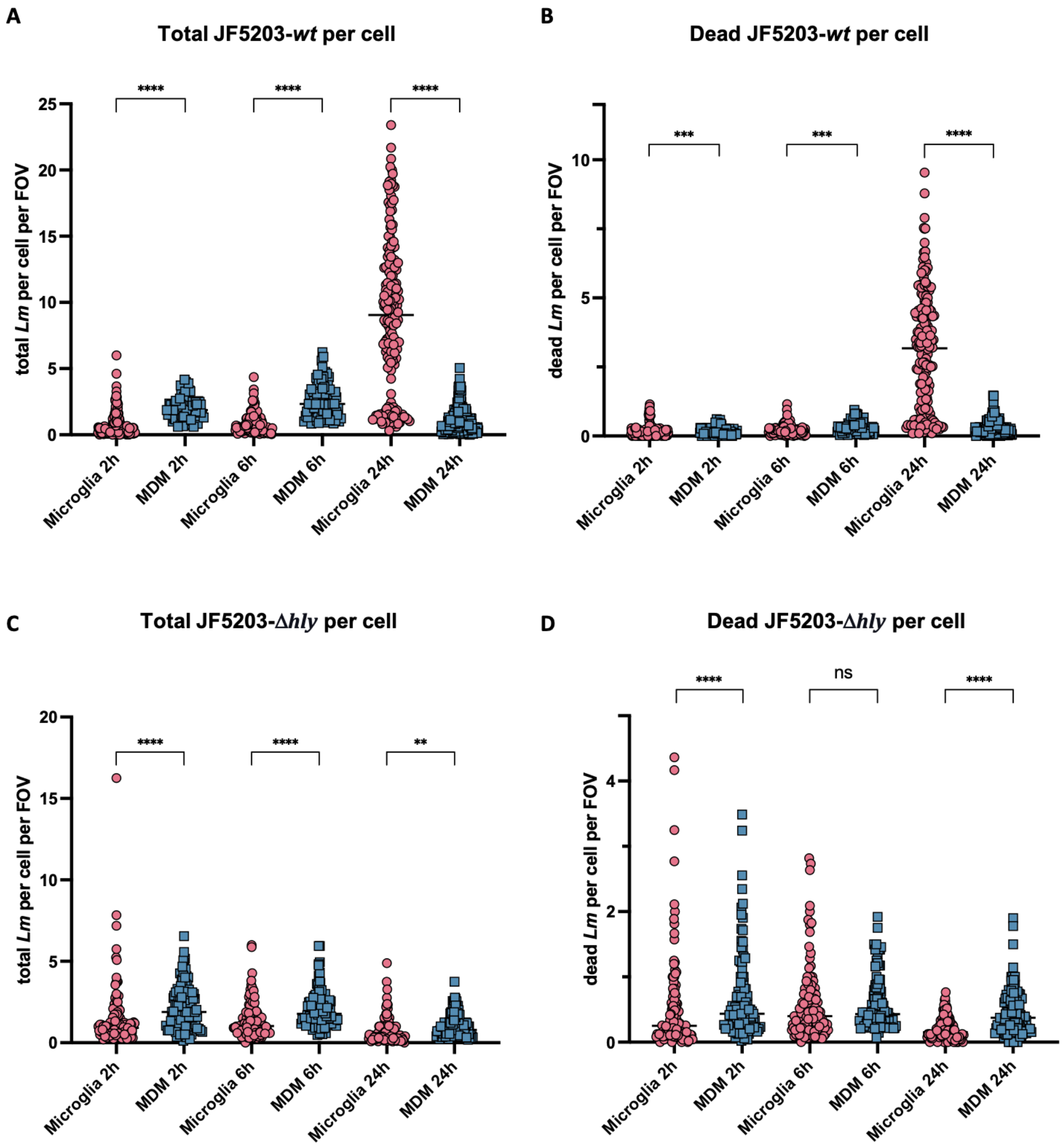

SI, Fig S10. Quantification of Baclight assay using JF5203-*wt* and JF5203-Δ*hly* taking into account all bacteria per field of view.

a, Total wt bacteria per cell; **b,** dead wt bacteria per cell; **c,** total Δ*hly* bacteria per cell; **d,** dead Δ*hly* bacteria per cell in microglia (pink) and monocyte-derived macrophages (blue) at 2h, 6h, and 24h post-infection. The bacterial quantifications are based on at least 3 biological replicates per cell type and condition, with 10 images analysed per time point and condition. Error bars indicate mean with SEM, ns: not significant, ** $p < 0.01$, *** $p < 0.001$ and **** $p < 0.0001$ (Mann-Whitney U test).

	Microglia	MDM	Total
**LLO**	6'034	3'502	9'536
**LC3b**	4'752	1'765	6'517
**Lamp1**	5'474	3'353	8'827
**Digitonin**	4'846	1'754	6'600
**Lysotracker**	8'231	2'581	10'812
**Actin**	32'745	17'078	49'823

SI, Table S1. Total number of bacterial counts in the image analysis of LLO, vacuolar markers (Lamp1, LC3b, Lysotracker), and cytosolic bacteria (digitonin, actin) in infected microglia and MDM.

	Sequence (restriction sites underlined)
**dInlA_1**	TICAGTCGACAAAAGAACTATCAACCAGCAACGC
**dInlA_2**	CTATTTACTTCGTTTTTTCTCACTATATACACTCC
**dInlA_3**	AGAAAACGAGTAAATAGAAGTAGTGTAAAGAGC
**dInlA_4**	TITTCCCGGGTTTAAATCCTTGAGCGAACTTAGG
**dInlB_1**	CCAAGTCGACTACAGCAACCTTGATGTTGATGG
**dInlB_2**	CTITCGTCCACACACTATCCTCTCCTTGATTCTAG
**dInlB_3**	GATAGTGTGTGGACGAAAGCTGCTAATTTAAGGG
**dInlB_4**	CTATCCCGGGTAGTGATGCTATCCACATTTTGGC

SI, Table S2. Sequences of primers used for the generation of *Listeria monocytogenes inlA* and *inlB* deletion mutants.

SI, Video S1. Live imaging of microglia and monocyte-derived macrophages (MDM) infected with GFP-expressing wild-type *Listeria monocytogenes* JF5203. This time-lapse video captures the behavior of microglia (00:00–00:15) and MDMs (00:15–00:30) over 35 minutes following bacterial exposure. Microglia appear stationary and take up individual bacteria, while MDMs display active phagocytosis, engulfing multiple bacteria and showing increased motility. Cells are labeled with Actin Tracker, and Hoechst 33342 is used to stain the nuclei.
